# The Molecular Genetic Architecture of Self-Employment

**DOI:** 10.1371/journal.pone.0060542

**Published:** 2013-04-04

**Authors:** Matthijs J. H. M. van der Loos, Cornelius A. Rietveld, Niina Eklund, Philipp D. Koellinger, Fernando Rivadeneira, Gonçalo R. Abecasis, Georgina A. Ankra-Badu, Sebastian E. Baumeister, Daniel J. Benjamin, Reiner Biffar, Stefan Blankenberg, Dorret I. Boomsma, David Cesarini, Francesco Cucca, Eco J. C. de Geus, George Dedoussis, Panos Deloukas, Maria Dimitriou, Guðny Eiriksdottir, Johan Eriksson, Christian Gieger, Vilmundur Gudnason, Birgit Höhne, Rolf Holle, Jouke-Jan Hottenga, Aaron Isaacs, Marjo-Riitta Järvelin, Magnus Johannesson, Marika Kaakinen, Mika Kähönen, Stavroula Kanoni, Maarit A. Laaksonen, Jari Lahti, Lenore J. Launer, Terho Lehtimäki, Marisa Loitfelder, Patrik K. E. Magnusson, Silvia Naitza, Ben A. Oostra, Markus Perola, Katja Petrovic, Lydia Quaye, Olli Raitakari, Samuli Ripatti, Paul Scheet, David Schlessinger, Carsten O. Schmidt, Helena Schmidt, Reinhold Schmidt, Andrea Senft, Albert V. Smith, Timothy D. Spector, Ida Surakka, Rauli Svento, Antonio Terracciano, Emmi Tikkanen, Cornelia M. van Duijn, Jorma Viikari, Henry Völzke, H. -Erich Wichmann, Philipp S. Wild, Sara M. Willems, Gonneke Willemsen, Frank J. A. van Rooij, Patrick J. F. Groenen, André G. Uitterlinden, Albert Hofman, A. Roy Thurik

**Affiliations:** 1 Department of Applied Economics, Erasmus School of Economics, Erasmus University Rotterdam, Rotterdam, The Netherlands; 2 Department of Epidemiology, Erasmus Medical Center, Rotterdam, The Netherlands; 3 Institute for Molecular Medicine Finland (FIMM), University of Helsinki, Helsinki, Finland; 4 Public Health Genomics Unit, Department of Chronic Disease Prevention, The National Institute for Health and Welfare (THL), Helsinki, Finland; 5 Department of Internal Medicine, Erasmus Medical Center, Rotterdam, The Netherlands; 6 Center for Statistical Genetics, Department of Biostatistics, University of Michigan School of Public Health, University of Michigan Ann Arbor, Michigan, United States of America; 7 Department of Twin Research and Genetic Epidemiology, King's College London, London, United Kingdom; 8 Institute for Community Medicine, University Medicine Greifswald, Greifswald, Germany; 9 Department of Economics, Cornell University, Ithaca, New York, United States of America; 10 Department of Prosthetic Dentistry, Gerodontology and Biomaterials, Centre of Oral Health, University of Greifswald, Greifswald, Germany; 11 Department of General and Interventional Cardiology, University Medical Center Hamburg-Eppendorf, Hamburg, Germany; 12 Netherlands Twin Register, Department of Biological Psychology, VU University Amsterdam, Amsterdam, The Netherlands; 13 Center for Experimental Social Science, Department of Economics, New York University, New York, New York, United States of America; 14 Istituto di Ricerca Genetica e Biomedica, Consiglio Nazionale delle Ricerche, Cagliari, Italy; 15 Department of Nutrition and Dietetics, Harokopio University of Athens, Athens, Greece; 16 Wellcome Trust Sanger Institute, Wellcome Trust Genome Campus, Hinxton, United Kingdom; 17 Icelandic Heart Association, Kopavogur, Iceland; 18 Diabetes Prevention Unit, Department of Chronic Disease Prevention, National Institute for Health and Welfare (THL), Helsinki, Finland; 19 Department of General Practice and Primary Health Care, University of Helsinki, Helsinki, Finland; 20 Folkhälsan Research Center, Helsinki, Finland; 21 Unit of General Practice, Helsinki University Central Hospital, Helsinki, Finland; 22 Vaasa Central Hospital, Vaasa, Finland; 23 Institute of Genetic Epidemiology, Helmholtz Zentrum München-German Research Center for Environmental Health, Neuherberg, Germany; 24 Department of Medicine, University of Iceland, Reykjavik, Iceland; 25 Institute of Epidemiology II, Helmholtz Zentrum München-German Research Center for Environmental Health, Neuherberg, Germany; 26 Institute of Health Economics and Health Care Management, Helmholtz Zentrum München-German Research Center for Environmental Health, Neuherberg, Germany; 27 Genetic Epidemiology Unit, Department of Epidemiology, Erasmus Medical Center, Rotterdam, The Netherlands; 28 Centre for Medical Systems Biology, Leiden, The Netherlands; 29 Institute of Health Sciences and Biocenter Oulu, University of Oulu, Oulu, Finland; 30 Department of Life Course and Services, National Institute for Health and Welfare, Oulu, Finland; 31 Department of Epidemiology and Biostatistics, MRC-HPA Centre for Environment and Health, Imperial College London, London, United Kingdom; 32 Department of Economics, Stockholm School of Economics, Stockholm, Sweden; 33 Department of Clinical Physiology, Tampere University Hospital, Tampere, Finland; 34 Department of Clinical Physiology, University of Tampere School of Medicine, Tampere, Finland; 35 Population Health Research Unit, Department of Health, Functional Capacity and Welfare, National Institute for Health and Welfare (THL), Helsinki, Finland; 36 Institute of Behavioural Sciences, University of Helsinki, Helsinki, Finland; 37 Laboratory of Epidemiology, Demography, and Biometry, Intramural Research Program, National Institute on Aging, Bethesda, Maryland, United States of America; 38 Department of Clinical Chemistry, Fimlab Laboratories, Tampere University Hospital, Tampere, Finland; 39 Department of Clinical Chemistry, University of Tampere School of Medicine, Tampere, Finland; 40 Division for Neurogeriatrics, Department of Neurology, Medical University of Graz, Graz, Austria; 41 Department of Medical Epidemiology and Biostatistics, Karolinska Institutet, Stockholm, Sweden; 42 Department of Clinical Genetics, Erasmus Medical Center, Rotterdam, The Netherlands; 43 Estonian Genome Center, University of Tartu, Tartu, Estonia; 44 Division of General Neurology, Department of Neurology, General Hospital and Medical University of Graz, Graz, Austria; 45 Department of Clinical Physiology and Nuclear Medicine, Turku University Hospital, Turku, Finland; 46 Research Centre of Applied and Preventive Cardiovascular Medicine, University of Turku, Turku, Finland; 47 Department of Epidemiology, The University of Texas MD Anderson Cancer Center, Houston, Texas, United States of America; 48 National Institute on Aging, National Institutes of Health, Baltimore, Maryland, United States of America; 49 Institute of Molecular Biology and Biochemistry, Medical University of Graz, Graz, Austria; 50 Institut für Medizinische Biometrie und Statistik, Universität zu Lübeck, Universitätsklinikum Schleswig-Holstein, Lübeck, Germany; 51 Department of Economics, Oulu Business School, University of Oulu, Oulu, Finland; 52 College of Medicine, Florida State University, Tallahassee, Florida, United States of America; 53 Department of Medicine, Turku University Hospital, Turku, Finland; 54 Department of Medicine, University of Turku, Turku, Finland; 55 Institute of Epidemiology I, Helmholtz Zentrum München-German Research Center for Environmental Health, Neuherberg, Germany; 56 Institute of Medical Informatics, Biometry and Epidemiology, Chair of Epidemiology, Ludwig-Maximilians-Universität, Munich, Germany; 57 Klinikum Grosshadern, Munich, Germany; 58 Center for Thrombosis and Hemostasis, University Medical Center Mainz, Johannes Gutenberg University Mainz, Mainz, Germany; 59 Department of Medicine 2, University Medical Center Mainz, Johannes Gutenberg University Mainz, Mainz, Germany; 60 Econometric Institute, Erasmus School of Economics, Erasmus University Rotterdam, Rotterdam, The Netherlands; 61 Panteia, Zoetermeer, The Netherlands; 62 GSCM-Montpellier Business School, Montpellier, France; University of Hong Kong, Hong Kong

## Abstract

Economic variables such as income, education, and occupation are known to affect mortality and morbidity, such as cardiovascular disease, and have also been shown to be partly heritable. However, very little is known about which genes influence economic variables, although these genes may have both a direct and an indirect effect on health. We report results from the first large-scale collaboration that studies the molecular genetic architecture of an economic variable–entrepreneurship–that was operationalized using self-employment, a widely-available proxy. Our results suggest that common SNPs when considered jointly explain about half of the narrow-sense heritability of self-employment estimated in twin data (*σ_g_* ^2^/*σ_P_* ^2^ = 25%, *h* ^2^ = 55%). However, a meta-analysis of genome-wide association studies across sixteen studies comprising 50,627 participants did not identify genome-wide significant SNPs. 58 SNPs with *p*<10^−5^ were tested in a replication sample (*n* = 3,271), but none replicated. Furthermore, a gene-based test shows that none of the genes that were previously suggested in the literature to influence entrepreneurship reveal significant associations. Finally, SNP-based genetic scores that use results from the meta-analysis capture less than 0.2% of the variance in self-employment in an independent sample (*p*≥0.039). Our results are consistent with a highly polygenic molecular genetic architecture of self-employment, with many genetic variants of small effect. Although self-employment is a multi-faceted, heavily environmentally influenced, and biologically distal trait, our results are similar to those for other genetically complex and biologically more proximate outcomes, such as height, intelligence, personality, and several diseases.

## Introduction

Economic variables such as income, education, and occupation are well-known to be related to health outcomes and longevity [1]–[10]. Specifically, there is a consistent inverse relation between indicators of socioeconomic status and cardiovascular disease [11]. For example, occupational choice is associated with the incidence of coronary heart disease among women [12]. Intriguingly, health outcomes, longevity, income, educational attainment, and occupational choice have all been shown to be partly heritable (see ref. [13] for complex diseases, refs. [14]–[17] for longevity, refs. [18]–[22] for education, refs. [23]–[25] for income, and refs. [26]–[28] for occupational choice). This suggests that the same genetic factors could be linked to socioeconomic status and health outcomes, or that indirect causal pathways from genetic variants to health outcomes exist that are mediated by individual behavior and the environment. For example, a potential mismatch between personal disposition and occupational choice may result in stress and decreased happiness, which have been shown to negatively affect (cardiovascular) disease incidence and longevity [29]–[32]. Therefore, knowledge about the specific molecular genetic architecture of socioeconomic variables and about the effects of mismatches between genetic predispositions and realized choices could yield important insights for epidemiology and public health policy. Unfortunately, most efforts to investigate the influence of genes on economic variables were until now limited to candidate gene studies that often failed to replicate later [33], [34].

This study reports results from the first large-scale collaboration that studies the molecular genetic architecture of a specific economic behavior–entrepreneurship–using data from high-density SNP arrays. Entrepreneurship has been associated with poor health [35], increased stress [36], relatively low average incomes [37], but also with greater job and life satisfaction [38]–[40]. The analysis of entrepreneurship is complicated by the fact that it is a multi-faceted phenomenon [41]. Individuals may engage in entrepreneurial activity for a variety of reasons. For example, certain individuals may be motivated to pursue a business opportunity or to gain independence, whereas others may do so because of unemployment and a lack of viable alternatives in paid employment. Despite this complexity, empirical evidence suggests that entrepreneurship tends to run in families [42]–[47], and recent twin studies consistently estimate the heritability of this behavior to be on the order of 50% [26]–[28]. As these results suggest that entrepreneurship is partly influenced by genetic variation, specific markers that are associated with entrepreneurship should, in principle, exist. Research that is aimed at discovering these specific markers has thus far been limited to one candidate gene study. This study [48] found evidence for an association between a specific genetic variant in the *DRD3* gene and entrepreneurship in a sample of *n* = 1,335. However, a more recent study [49] failed to replicate this association in three larger samples of *n* = 5,374, *n* = 2,066, and *n* = 1,925.

The molecular genetic architecture of entrepreneurship therefore remains largely unknown. A variety of alternative architectures could account for heritable variation. For example, there may be a small number of rare variants with strong effects, multiple common variants with small or modest effects, or some combination of these possibilities [50], [51]. Therefore, we aimed to identify the molecular genetic architecture of entrepreneurship to facilitate a more sophisticated understanding of the nature of the associated heritable variation.

We use self-employment as a proxy for entrepreneurship in this study, which is the most widely available proxy for entrepreneurship. Self-employment is defined as having started, owned, and managed a business. Initially, we used a classical twin design to estimate the heritability of the tendency to engage in self-employment. We performed this analysis to determine the comparability of our results with (1) estimates of previous twin studies, and (2) estimates from a novel method from molecular genetics. This recently described method [52] is used here to quantify the proportion of variance that is explained by common SNPs (and unknown causal variants that are in linkage disequilibrium with these SNPs) in the tendency to engage in self-employment.

Furthermore, we performed a meta-analysis of genome-wide association studies (GWASs) of self-employment from sixteen studies to identify genetic variants that are robustly associated with self-employment. Together, these studies comprised 50,627 participants of European ancestry who are part of the Gentrepreneur Consortium [53], [54]. This study is the first large-scale effort to identify common genetic variants that are associated with an economic variable. We also tested whether self-employment could be predicted out-of-sample solely using genotype data and the results of our meta-analysis.

Theoretical and empirical evidence from entrepreneurship research suggests that there may be differences between males and females with respect to the type of businesses they start. These differences also extend to individuals' motivations, goals, and resources [55]–[59] and exist because women face different–and typically more–barriers to entrepreneurship than men [60]–[62]. Therefore, we performed both pooled and sex-stratified analyses for all of our investigations.

## Materials and Methods

### Participating studies and self-employment measures

The analyses were performed within the Gentrepreneur Consortium [53], [54], which included two out of the five studies that participate in the Cohorts for Heart and Aging Research in Genomic Epidemiology (CHARGE) Consortium [63] and fourteen additional studies. The discovery studies included the Age, Gene/Environment Susceptibility–Reykjavik Study (AGES), the Austrian Stroke Prevention Study (ASPS), the Erasmus Rucphen Family study (ERF), the Gutenberg Health Study (GHS), Health 2000 (H2000), the Helsinki Birth Cohort Study (HBCS), the Health and Retirement Study (HRS), the Cooperative Health Research in the Region of Augsburg (KORA S4), the Northern Finland Birth Cohort 1966 (NFBC1966), the Netherlands Twin Register Cohort 1 (NTR1), the Netherlands Twin Register Cohort 2 (NTR2), the Rotterdam Study Baseline (RS-I), the Rotterdam Study Extension of Baseline (RS-II), the Rotterdam Study Young (RS-III), the SardiNIA Study of Aging (SardiNIA), the Study of Health in Pomerania (SHIP), The Hellenic study of Interactions between SNPs & Eating in Atherosclerosis Susceptibility (THISEAS), the UK Adult Twin Registry (TwinsUK), and the Cardiovascular Risk in Young Finns Study (YFS). The Swedish Twin Registry (STR) served as an *in silico* replication study, as genome-wide data were only available following the completion of the discovery stage.

The studies collected data regarding occupational status using questionnaires or interviews, from which self-employment status was distilled. Self-employment measures were defined in collaboration with the consortium leaders to minimize heterogeneity across participating studies. The cases were defined as individuals who were self-employed at least once, and the controls were defined as individuals who were never self-employed during their working life. However, for a number of studies, reliable data regarding work-life history were unavailable, possibly resulting in the inclusion of previously self-employed individuals in the control group. The details regarding the background and self-employment measures of each of the discovery studies and of the replication study are given in Table S1.

### Ethics statement

All participating studies were approved by the relevant institutional review boards or the local research ethics committees, including the Icelandic National Bioethics Committee (VSN: 00-063), the Icelandic Data Protection Authority, and the Institutional Review Board for the National Institute on Aging (AGES); the Ethics Committee of the Medical Faculty of the University of Graz (ASPS); the Medical Ethics Committee at Erasmus University which approved the protocols for the ascertainment and examination of human subjects (ERF); the local ethics committee and data safety commissioner, the sampling design was approved by the federal data safety commissioner (GHS); the Ethics Committee for Epidemiology and Public Health in the Hospital District of Helsinki and Uusimaa in Finland, in accordance with the ethical standards of the Declaration of Helsinki (H2000); the Ethics Committee of Epidemiology and Public Health of the Hospital District of Helsinki and Uusimaa (HBCS); the Health Sciences Institutional Review Board at the University of Michigan (HRS); the Ethics Committee of the Bavarian Medical Association (KORA S4); the Ethics Committee of the University Hospital of Oulu (NFBC1966); the VU University Medical Ethical Committee (NTR); the Medical Ethics Committee of the Erasmus Medical Center (RS); the local Ethics Committee for the Istituto di Ricerca Genetica e Biomedica, Consiglio Nazionale delle Ricerche and the MedStar Research Institute, responsible for intramural research at the National Institute of Aging (SardiNIA); the Ethics Committee of the University of Greifswald (SHIP), the Ethical Review Board in Stockholm (STR); the Bioethics Committee of the Harokopio University of Athens (THISEAS); the NRES Committee London-Westminster (TwinsUK); the local Ethics Committees of the participating universities (YFS). Written informed consent was provided by all of the participants.

### Genotyping, imputation, and quality control

The seventeen participating studies used a variety of commercially available SNP genotyping platforms to genotype their participants. Each study performed quality control of their genotypic data and imputed the genotypes of each participant to a common set of approximately 2.5 million SNPs from the HapMap CEU population. The exceptions to this were THISEAS, which only supplied results for directly genotyped SNPs, and HRS, which imputed to the 1,000 Genomes Project Phase I v3 panel. Prior to the meta-analysis, we performed parallel quality control of the association results for each study. SNPs were excluded on the basis of minor allele frequency (MAF<0.01 or MAF<0.05 if deemed necessary) and if the imputation quality (a measure of the observed variance divided by the expected variance of the imputed allele dosage from the imputation software output) was less than 0.4. Following these exclusions, approximately 2.4 million SNPs remained. Study-specific details regarding the genotyping, imputation, and quality control are given in Table S2.

### Statistical analysis

Tetrachoric correlations were used to calculate self-employment correlations for MZ and DZ twin pairs. This analysis assumes a latent normally distributed tendency to engage in self-employment. We estimated the heritability of the tendency to engage in self-employment in the replication study using standard twin study methods, which were implemented in the program Mx [64]. Only complete twin pairs with data regarding self-employment status were included in the analysis and opposite-sex DZ twin pairs were excluded, resulting in a final sample size of 4,464 individuals. Specifically, for pooled males and females, males only, and females only, we fitted the three following nested models using the maximum likelihood approach on the raw data: (1) a model including an additive genetic effect, a shared common environment effect, and an individual-specific environment effect (the *ACE* model); (2) a model that included only an additive genetic and an individual-specific environment effect (the *AE* model); and (3) a model including only a common environment effect and an individual-specific environment effect (the *CE* model). For all of the samples, we controlled for a *z*-score of age by estimating age-specific thresholds. For the pooled sample, we additionally controlled for sex in a similar way.

We used the method that was recently developed by Yang et al. [52] to estimate the proportion of variance in the tendency to engage in self-employment that is explained by all of the common genotyped SNPs. The method is implemented in the GCTA software [65] and hinges on the assumption that in a sample of unrelated individuals, environmental factors segregate independently in the pedigree from the degree of genetic relatedness. In contrast to the twin study design, genetic relatedness is not inferred from the pedigree but is estimated directly from genome-wide SNP data. Under the assumption of no confounding by environmental variables, we can then estimate the accounted-for variance by relating the estimated genetic relatedness between pairs of individuals to their phenotypic correlation. The resulting estimate is actually a lower bound of the heritability that is estimated from classic twin and family studies. The reason for this is that twin and family studies capture the variation that is due to all of the additive causal variants, whereas the more recently developed method only captures the variants that are either directly genotyped or in linkage disequilibrium.

We used a combined sample of individuals from one of the discovery studies (RS-I) and the replication study (STR) to estimate the accounted-for variance. We restricted the sample from each study to individuals for whom data regarding self-employment were available. Additionally, we included only one randomly selected individual from each family in the STR sample. A second round of quality control of the genotypic data was then performed for both studies. In the RS-I sample, we excluded 3,748 SNPs because they failed a test of Hardy-Weinberg equilibrium at *p*<1×10^−6^. We removed 24,993 SNPs with minor allele frequencies that were lower than 0.01 and another 6,665 due to data missingness greater than 5%. In total, 5,374 individuals and 561,466 autosomal SNPs were included in the analysis. In the STR sample, we removed two SNPs because they failed a test of Hardy-Weinberg equilibrium at *p*<1×10^−6^. Another 628 SNPs with a minor allele frequency lower than 0.01 were removed, as were two SNPs with data missingness greater than 5%. Therefore, 643,924 autosomal SNPs and 2,589 individuals were included in the analysis.

We then estimated the genetic relationships among 7,963 individuals in the combined sample from the 301,115 common autosomal SNPs. We dropped one of any pair of individuals with an estimated genetic relationship that was >0.025 while maximizing the remaining sample size to exclude the possibility of ascribing shared environmental effects to genetic effects and/or including the effects of causal variants not correlated with the genotyped SNPs but captured by the pedigree. The maximum relatedness in the remaining sample of 6,223 individuals therefore approximately corresponds to cousins two to three times removed [52].

Next, the linear mixed model *y* = *μ*+*g*+*e* was fitted, where *y* is the binary phenotype, *g* the total additive genetic effect of the SNPs, and *e* is a residual effect. The restricted maximum likelihood (REML) was used to estimate the variance of the total additive genetic effect *σ_g_* ^2^ of the SNPs by fitting the genetic relationships as the covariance structure. Because the analyzed phenotype is binary, *σ_g_* ^2^ is the variance of the total additive genetics effects on the observed 0–1 scale. A latent normally distributed tendency to engage in self-employment was assumed when transforming the explained variance from the observed 0–1 scale to the latent scale using the transformation that is derived in the appendix of Dempster and Lerner [66]. For all of the analyses, we controlled for a *z*-score of age, study, and the first ten principal components of the genetic relationships of the combined sample. In the pooled sample, we also controlled for sex.

In addition to the Yang et al. [52] method, we employed a novel method developed by So et al. [67] that serves the same purpose, i.e., estimating the proportion of variance in the tendency to engage in self-employment that is explained by all of the common SNPs. However, in contrast to the Yang et al. [52] method, So et al.'s method does not require raw genotype data but attempts to recover the accounted-for variance from the meta-analysis results. Using PLINK [68], we restricted the meta-analysis results to SNPs that were present in the HapMap Phase II CEU panel (release 23a) and pruned those in strong linkage disequilibrium with other SNPs using a pairwise *r* ^2^ threshold of 0.25 in a window of 100 SNPs that slides in 25 SNP increments. After this procedure 172,742, 175,970, and 172,989 SNPs remained in the pooled males and females, males only, and females only sample, respectively. We used the Gaussian Kernel function, considered under the null-hypothesis of no association, and ran the simulation 500 times in each sample.

The genome-wide association analysis of self-employment was independently performed by each study according to a predefined analysis plan. The analyses were performed for pooled males and females, males only, and females only using an additive genetic model, controlling for age (≤29 [reference]; 30–39; 40–49;≥50) and sex in the pooled sample. To control for population stratification, the first four principal components of the genotypic data were also included if available. We provide details regarding the statistical analysis within each study in Table S2.

Following the association analyses, the genomic inflation factor *λ* was calculated for each sample to quantify any remaining population stratification or cryptic relatedness. The lowest inflation factor was 0.989, and the highest was 1.156, although this latter value was for a study that did not include the first four principal components of the genotypic data in the analysis (Table S3). Genomic control [69] was applied in samples with inflation factors that were greater than one by adjusting the test statistics.

We next performed fixed-effect meta-analyses of the association results from the discovery studies for pooled males and females, males only, and females only using METAL software [70]. Although the phenotype was defined as self-employment in each participating study, we could not harmonize the exact wording of the question on which the self-employment measure was based. In addition, the connotations of self-employment may depend to some extent on the level of economic development and culture. This may lead to unobserved gene-environment interactions that could introduce additional noise in the GWAS results pooled across studies. We combined the association results using weighted *z*-scores that were based on the *p*-values and the direction of the effects. This method first computes a per-study signed *z*-score for each SNP based on its *p*-value and the effect direction. The *z*-scores are then summed with weights that are proportional to the square root of the sample size of each study. Following the meta-analyses, only autosomal SNPs that were present in the Hapmap Phase II CEU panel (release 22, NCBI build 36) and in at least half of the contributing samples in each meta-analysis were retained prior to both reporting *p*-values and the creation of the Q–Q and Manhattan plots. We *a priori* set the genome-wide significance threshold to *p*<5×10^−8^. SNPs with *p*<1×10^−5^ were considered suggestive and also carried forward to the replication stage. The heterogeneity of the test statistics between the studies was assessed using the *I* ^2^ metric [71], [72] and Cochran's *Q* statistic [73].

Replication was attempted for significant and suggestive SNPs from each meta-analysis using an *in silico* replication study comprising 3,271 individuals. The association results for these SNPs were looked up in the replication study and meta-analyzed together with the discovery samples for pooled males and females, males only, and females only. To adjust for family relationships in the replication study, we performed family-based association tests implemented in the MERLIN software [74].

We used the discovery meta-analyses results to calculate gene-based *p*-values using the VEGAS program [75]. The positions of the UCSC Genome Browser hg18 assembly were employed to assign SNPs to genes, which included regions that were ±50 kb from the 5′ and 3′ UTRs.

For the prediction analyses, we followed the approach that was pioneered by The International Schizophrenia Consortium [76] and used the association results from the discovery meta-analyses to predict self-employment in the STR. Specifically, twelve overlapping sets of SNPs that were nominally associated in the discovery meta-analyses were created for different significance thresholds (*p*_T_<0.01, *p*_T_<0.05, *p*_T_<0.1, *p*_T_<0.2, *p*_T_<0.3, *p*_T_<0.4, *p*_T_<0.5, *p*_T_<0.6, *p*_T_<0.7, *p*_T_<0.8, *p*_T_<0.9, and *p*_T_≤1). These sets were used as inputs for score calculation in the STR. We restricted the STR sample to individuals for whom data regarding self-employment were available and included only one randomly selected individual from each family, resulting in a final sample size of 2,589 individuals for the prediction analyses.

Prior to calculating the scores for each individual in the STR, we followed [76] and selected all of the autosomal SNPs, pruning those in strong linkage disequilibrium with other SNPs. This process was performed using a pairwise *r* ^2^ threshold of 0.25 in a window of 200 SNPs that slides in five SNP increments. Following this exclusion process, 135,823 SNPs remained. The PLINK [68] ‘score’ function was then used to calculate the total score for each individual in the STR. The score is defined as the sum of the number of score alleles, weighted by the estimated coefficients from the discovery meta-analyses, divided by the number of non-missing genotypes. If an individual was missing a genotype, it was imputed as the mean genotype based on the score allele frequency in the STR. On average, the score was calculated from approximately 120,000 SNPs given that (1) the coefficients were only estimated for SNPs in the HapMap CEU population in the discovery meta-analyses, and (2) the overlap with the genotyped SNPs was not perfect. Lastly, we regressed self-employment onto the score using a logistic regression model. The variance that was explained by the score was estimated using the Nagelkerke pseudo-*R* ^2^ of the fitted model. We also calculated the area under the receiver operating characteristic curve (AUC) to evaluate the prediction accuracy.

## Results

### Heritability of self-employment and the degree of variance that is accounted for by common SNPs

We used data from the Swedish Twin Registry (STR) and the classical twin design to estimate the heritability of the tendency to engage in self-employment. We computed the tetrachoric correlations between the tendencies to engage in self-employment within monozygotic (MZ) and dizygotic (DZ) twin pairs. Table 1 indicates that the correlations within the MZ twin pairs were consistently higher than within the DZ twin pairs for males only, for females only, and for pooled males and females. We note that the correlation within DZ twin pairs in the pooled sample was higher than for the DZ correlations in males and females when the two sexes are considered separately. This effect most likely results from imprecise estimation of the tetrachoric correlations due to the small number of cases. When we computed Pearson correlations, the pooled DZ twin pairs correlation was in between the male and female DZ twin pairs correlations. Applying Falconer's formula [77] to the correlations in Table 1, yields *h* ^2^ estimates of 0.39 for pooled males and females, 0.69 for males only, and 0.34 for females only.

**Table 1:** Tetrachoric correlations in the tendency to engage in self-employment for MZ and DZ twin pairs in STR for pooled males and females, males only, and females only.

	Pooled		Males		Females	
	MZ	DZ	MZ	DZ	MZ	DZ
*n*	1,062	1,170	419	469	643	701
Concordant pairs	839	868	320	307	519	561
Discordant pairs	223	302	99	162	124	140
Pairwise concordance (%)	79.0	74.2	76.4	65.5	80.7	80.0
Tetrachoric *ρ*	0.560	0.363	0.677	0.332	0.401	0.230
s.e.	0.042	0.052	0.053	0.072	0.078	0.090

*n* refers to the number of twin pairs; s.e.: standard error.

A maximum likelihood approach was employed to estimate the relative contributions of the additive genetic (*A*), shared common environment (*C*), and individual-specific environment (*E*) components. This approach was performed using an *ACE* model and two nested submodels for pooled males and females, males only, and females only. Table 2 gives the estimates of the *A* component as 0.54 for pooled males and females, 0.67 for males only, and 0.38 for females only. The estimates of the *C* component were 0.01 for pooled males and females, 0.00 for males only, and 0.02 for females only. The *A* component was significant at the 95% confidence level for pooled males and females, and for males only, although the confidence intervals were very wide. This component was not significant for the females only analysis. However, the *χ* ^2^ test for goodness-of-fit and Akaike information criterion indicated that the *AE* model was the best-fitting model in all samples. In this submodel, the estimate for the *A* component for females only did not change markedly compared to the *ACE* model but was significant at the 95% confidence level. The estimates of the *A* component for pooled males and females, and males only were 0.55 and 0.67, respectively; these results were significant.

**Table 2:** Results of fitting *ACE*, *AE*, and *CE* models to the tendency to engage in self-employment in STR for pooled males and females, males only, and females only.

Sample	Model	*A*	(95% CI)	*C*	(95% CI)	*E*	(95% CI)	*χ* ^2^	*p*-value	AIC
Pooled	*ACE*	0.54	(0.25–0.63)	0.01	(0.00–0.25)	0.45	(0.37–0.55)	–	–	−4,707.96
	*AE*	0.55	(0.46–0.63)	–	–	0.45	(0.37–0.54)	0.01	0.929	−4,709.95
	*CE*	–	–	0.42	(0.35–0.49)	0.58	(0.51–0.65)	13.60	<0.001	−4,696.36
Males	*ACE*	0.67	(0.33–0.76)	0.00	(0.00–0.28)	0.33	(0.24–0.44)	–	–	−1,417.15
	*AE*	0.67	(0.56–0.76)	–	–	0.33	(0.24–0.44)	0.00	1.000	−1,419.15
	*CE*	–	–	0.50	(0.41–0.59)	0.50	(0.41–0.59)	14.27	<0.001	−1,404.88
Females	*ACE*	0.38	(0.00–0.53)	0.02	(0.00–0.38)	0.60	(0.47–0.76)	–	–	−3,276.62
	*AE*	0.40	(0.26–0.53)	–	–	0.60	(0.47–0.75)	0.01	0.919	−3,278.61
	*CE*	–	–	0.31	(0.19–0.42)	0.69	(0.58–0.81)	2.50	0.114	−3,276.12

For pooled males and females the analyses are based on 2,232 twin pairs (1,062 MZ and 1,170 DZ), for males only on 888 twin pairs (419 MZ and 469 DZ), and for females only on 1,344 twin pairs (643 MZ and 701 DZ). The share of self-employed was 21% for the pooled, 32% for the male, and 13% for the female sample. In all samples we controlled for age and in the pooled sample for sex; *A*: additive genetic component; *C*: shared common environment component; *E*: individual-specific environment component; 95% CI: 95% confidence interval; *χ* ^2^: *χ* ^2^ test for goodness-of-fit, the baseline model is the *ACE* model; AIC: Akaike information criterion.

The recently developed method by Yang et al. [52] was employed to estimate the degree of variance in the tendency to engage in self-employment that is explained by all of the genotyped autosomal SNPs in the GWAS datasets. The proportion of the explained variance was estimated for pooled males and females, males only, and females only. To maximize the power of the analysis, we used a combined sample of one of the discovery studies (Rotterdam Study Baseline [RS-I]) and the STR. We estimated that 25% (*p* = 0.032) of the variance in the tendency to engage in self-employment could be explained by the common genotyped autosomal SNPs for pooled males and females (Table 3). The variance that could be explained for males only and for females only was 25% (*p* = 0.152) and 0% (*p* = 0.499), respectively. The estimates for males and females separately were not significantly different from one other. The fact that the variance that is explained was zero for females is most likely due to the very low number of female cases (*n* = 353) compared to the number of controls (*n* = 3,482). The estimation of the explained variance is therefore very imprecise. We also estimated the variance that was explained for pooled males and females, males only, and females only in the RS-I and the STR separately. The estimates were not significant because the standard errors of these estimates depend heavily on the sample size. However, considered in their entirety, the results were consistent with the estimates that we present for the combined RS-I and STR samples. Overall, the results for pooled males and females and for males indicated that the degree of variance in the tendency to engage in self-employment that is explained by all of the common autosomal SNPs simultaneously is only approximately half of the narrow-sense heritability that is estimated using the STR and the classical twin design. Furthermore, estimates using the method developed by So et al. [67] also provide non-zero estimates for heritability. Specifically, the accounted-for variance was 7% for pooled males and females, 21% for males only, and 15% for females only. However, confidence intervals and standard errors could not be calculated for these estimates because not all raw genotype data were available, prohibiting further interpretation of these results.

**Table 3:** Variance in the tendency to engage in self-employment explained by all autosomal SNPs in a combined sample of RS-I and STR for pooled males and females, males only, and females only.

Sample	*σ_g_* ^2^/*σ_P_* ^2^	s.e.	*p*-value	*n*	Cases	(%)	Controls	(%)
Pooled	0.25	0.14	0.032	6,223	905	(14.5)	5,318	(85.5)
Males	0.25	0.24	0.152	2,986	618	(20.7)	2,368	(79.3)
Females	0.00	0.28	0.499	3,835	353	(9.2)	3,482	(90.8)

The genetic relationships were estimated from 301,115 directly genotyped autosomal SNPs that were available in both studies. All analyses controlled for age, study, and the first 10 principal components of the genetic similarity matrix of the combined sample of RS-I and STR. In the pooled sample we also controlled for sex. The results did not change markedly when 4 or 20 principal components were included; *σ_g_* ^2^/*σ_P_* ^2^: proportion of phenotypic variance explained by the variance of the total additive genetic effects of the 301,115 autosomal SNPs; s.e.: standard error; *p*-value: *p*-value from a likelihood ratio (LR) test assuming that the LR is distributed as a 50∶50 mixture of zero and *χ*_1_^2^.

### Meta-analyses of genome-wide association studies

We performed genome-wide association analyses of self-employment using the data from sixteen discovery studies. These studies comprised 7,734 participants who had been self-employed at least once and 42,893 participants who did not report being self-employed. Table 4 includes the descriptive statistics for the studies. The mean ages in the pooled samples of males and females ranged from 31 to 68.8 years, and the average age across all of the studies was 53.4 years. Following independent association analyses for each study, we performed a fixed-effect meta-analysis of the study-level results for approximately 2.4 million SNPs using a pooled *z*-score approach.

**Table 4:** Descriptive statistics of the sixteen discovery studies and the replication study.

	Pooled		Males		Females		Demographics	
Study	Cases	Controls	Cases	Controls	Cases	Controls	Mean age	SD age
AGES	529	2,690	439	913	90	1,777	51.2	6.5
ASPS	46	788	26	336	20	452	65.2	8.1
ERF	214	857	113	366	101	491	47.2	13.4
GHS	424	2,706	282	1,332	142	1,374	55.9	10.9
H2000	228	1,895	145	890	83	1,005	50.7	11.1
HBCS	265	1,459	141	595	124	864	61.5	2.9
HRS	1947	4273	1048	1780	899	2493	63.6	7.9
KORA S4	177	1,588	121	760	56	828	53.8	8.8
NFBC1966	462	3,772	322	1,718	140	2,054	31.0	0.0
NTR1	201	1,354	94	494	107	860	46.4	13.3
NTR2	166	818	77	355	89	463	51.0	13.8
RS-I	531	4,843	319	1,994	212	2,849	68.8	8.8
RS-II	197	1,869	113	848	84	1,021	64.8	8.0
RS-III	209	1,716	138	746	71	970	56.1	5.8
SardiNIA	740	3,402	515	1,207	225	2,195	46.3	17.1
SHIP	157	3,906	107	1,891	50	2,015	49.7	16.3
THISEAS	204	481	176	243	28	238	51.1	11.2
TwinsUK^a^	822	2,333	–	–	730	2,165	54.5	12.4
YFS	215	2,143	89	1,194	126	949	37.6	5.0
Total discovery	7,734	42,893	4,265	17,662	3,377	25,063	53.4	9.4
STR	737	2,534	484	925	253	1,609	60.6	4.3
Total combined	8,471	45,427	4,749	18,587	3,630	26,672	53.8	9.1

AGES: Age, Gene/Environment Susceptibility–Reykjavik Study; ASPS: Austrian Stroke Prevention Study; ERF: Erasmus Rucphen Family study; GHS: Gutenberg Health Study; H2000: Health 2000; HBCS: Helsinki Birth Cohort Study; HRS: Health and Retirement Study; KORA S4: Cooperative Health Research in the Region of Augsburg; NFBC1966: Northern Finland Birth Cohort 1966; NTR1: Netherlands Twin Register Cohort 1; NTR2: Netherlands Twin Register Cohort 2; RS-I: Rotterdam Study Baseline; RS-II: Rotterdam Study Extension of Baseline; RS-III: Rotterdam Study Young; SardiNIA: SardiNIA Study of Aging; SHIP: Study of Health in Pomerania; THISEAS: The Hellenic study of Interactions between SNPs & Eating in Atherosclerosis Susceptibility; TwinsUK: the UK Adult Twin Registry; YFS: the Cardiovascular Risk in Young Finns Study; STR: Swedish Twin Registry; Cases: number of participants that were at least once self-employed; Controls: number of participants that were not, and ideally never, self-employed; SD: standard deviation.

a The number of male participants was insufficient for a male stratified analysis.

**Figure 1:**
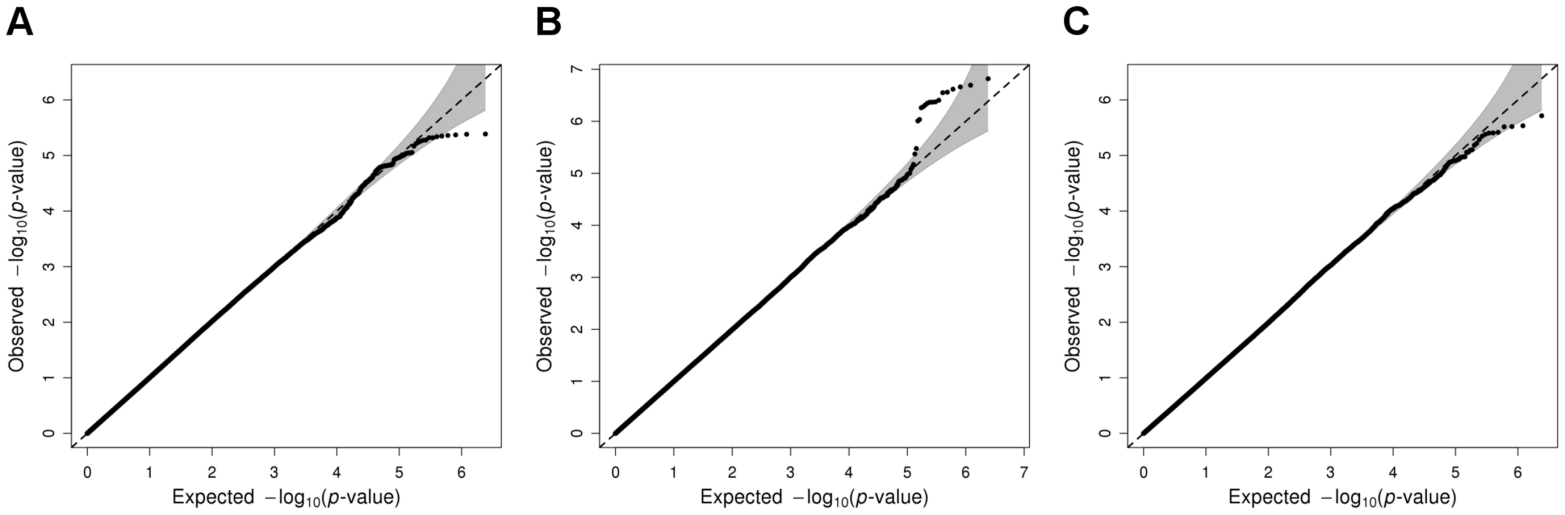
Q–Q plots of the self-employment discovery meta-analyses. Q–Q plot of the self-employment discovery meta-analysis for (A) pooled males and females, (B) males only, and (C) females only. The grey shaded areas in the Q–Q plots represent the 95% confidence bands around the *p*-values.

**Figure 2:**
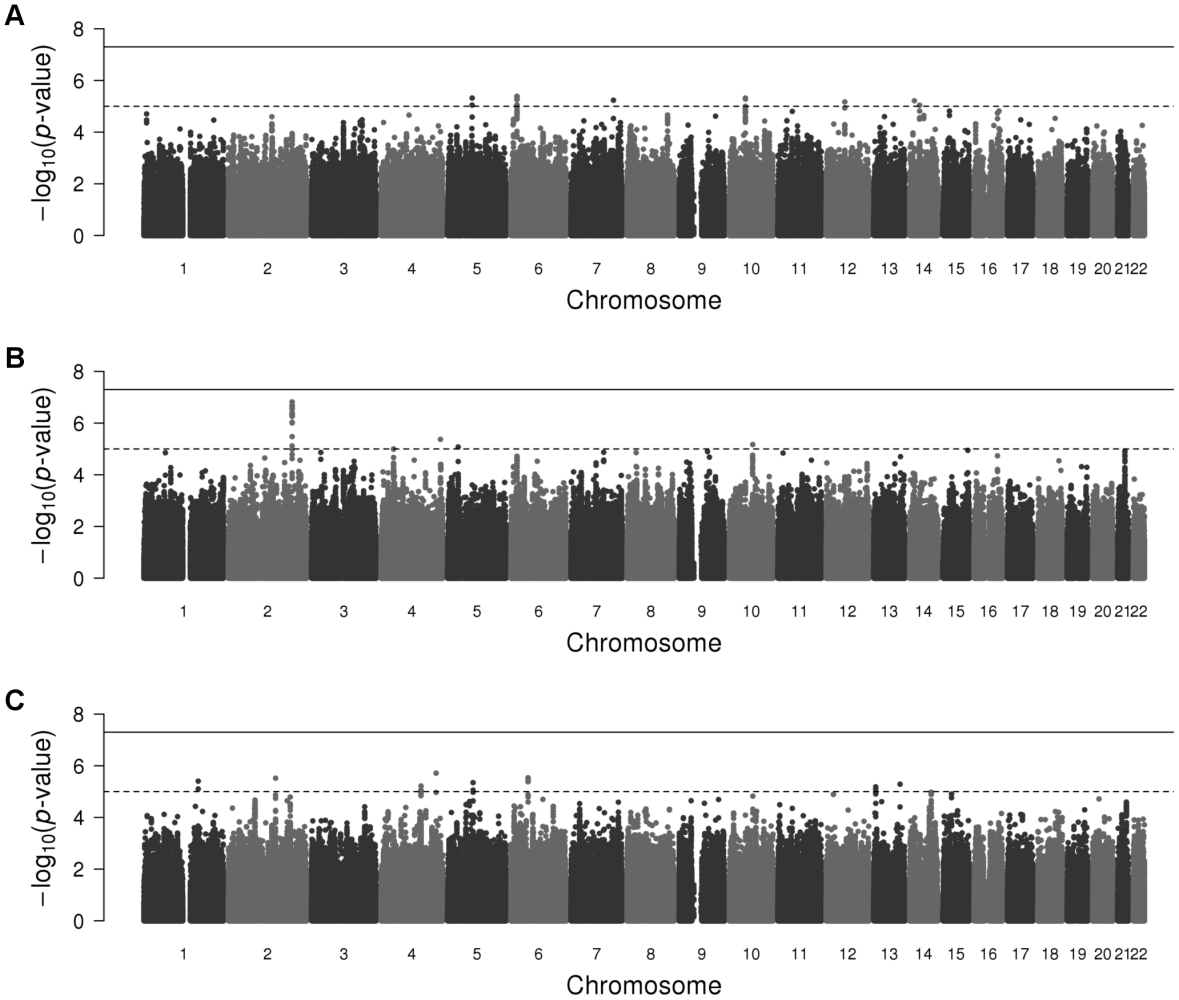
Manhattan plots of the self-employment discovery meta-analyses. Manhattan plot of the self-employment discovery meta-analysis for (A) pooled males and females, (B) males only, and (C) females only. SNPs are plotted on the *x*-axis according to their position on each chromosome against association with self-employment on the *y*-axis (shown as −log10 *p*-value). The solid line indicates the threshold for genome-wide significance (*p*<5×10^−8^) and the dashed line the threshold for suggestive SNPs (*p*<1×10^−5^).

The discovery meta-analysis Q–Q plot (Figure 1A) did not indicate a strong deviation for the lowest *p*-values. However, no confounding issues related to population stratification, cryptic relatedness, or genotyping errors were detected, as no systematic deviation from the expectation under the null hypothesis of no association was observed [78]. As illustrated in the Manhattan plot (Figure 2A), we observed twenty SNPs with 4.1×10^−6^≤*p*<1×10^−5^ (Tables 5 and S4). The SNP with the lowest *p*-value, rs6906622 (*p* = 4.10×10^−6^), was located near the *RNF144B* gene, with most studies indicating that the minor allele increased the probability of being self-employed (Table 5).

**Table 5:** Top SNPs (*p*<1×10^−5^) from the self-employment discovery meta-analyses for pooled males and females, males only, and females only.

SNP	Chr.	Pos.	Effect /non-effect allele	EAF	*p*-value	Direction	Nearest gene	Number of SNPs in region
Pooled								
rs6906622	6	18,596,287	T/C	0.21	4.10×10^−6^	++−++++++++−++++?++	*RNF144B*	12
rs2358531	5	75,515,542	A/G	0.71	4.79×10^−6^	−−−?−−−−−−+−−+−−?−−	*SV2C*	2
rs10776614	10	49,433,172	T/C	0.16	4.79×10^−6^	−+−−−−−+−−−−−−−−?−−	*ARHGAP22*	2
rs17166082	7	131,363,900	A/G	0.06	5.82×10^−6^	−?−?−−?−−+−−−−−−?−−	*PLXNA4*	1
rs994208	14	33,531,622	C/G	0.66	6.11×10^−6^	−+−−−−−−−−−−−−−−?−−	*EGLN3*	1
rs3847697	12	57,282,257	T/C	0.44	6.79×10^−6^	−−+−−−−−?−+−+−?−?−−	*LRIG3*	1
rs3742467	14	49,709,284	T/C	0.88	9.11×10^−6^	+++++−?+−+++−+++?++	*SOS2*	1
Males								
rs6738407	2	196,851,876	A/G	0.20	1.52×10^−7^	−−−−−−−−−+−−−−−−?−	*HECW2*	18
rs6825440	4	183,636,063	A/T	0.24	4.25×10^−6^	−+−−−−−−−−+−−−+−?−	*ODZ3*	1
rs7904494	10	72,056,694	A/T	0.78	6.74×10^−6^	+−+−−−?−−++−−−−−?−	*PRF1*	1
rs4867424	5	32,331,331	T/C	0.49	8.39×10^−6^	−−+−−−−−−−−−−−−−?−	*MTMR12*	1
rs2712008	4	38,752,396	T/G	0.14	9.94×10^−6^	+−++++?+++++−+++?+	*KLHL5*	1
Females								
rs2331548	4	170,199,179	A/G	0.96	1.93×10^−6^	??+?++++++++++++?++	*CBR4*	1
rs521326	6	52,927,336	A/G	0.61	2.92×10^−6^	−−−−−−−−−−−−+−−−?−−	*GSTA4*	5
rs1022335	2	145,813,253	A/T	0.37	3.02×10^−6^	−−−−−−?−−−−−+−−−?−−	*ZEB2*	1
rs10753804	1	168,583,032	T/C	0.49	3.92×10^−6^	−−−−−−?−−−−+−−−−?−−	*SCYL1BP1*	2
rs562487	5	78,442,190	A/G	0.48	4.49×10^−6^	+++++−++−+−++−++?++	*BHMT*	2
rs9557259	13	99,031,403	T/C	0.06	5.16×10^−6^	??−?++?++++++?????+	*TM9SF2*	1
rs1383043	4	123,562,066	A/G	0.38	6.05×10^−6^	−−+−−−−+−−−−−−−−??+	*ADAD1*	2
rs9578700	13	23,775,308	A/G	0.67	6.53×10^−6^	−+++−−−−−−−−−−−−?−+	*SPATA13*	2

Chr.: chromosome; Pos.: position; EAF: average effect allele frequency; In the column “direction”, the studies are in the following order: 1. AGES, 2. ASPS, 3. ERF, 4. GHS, 5. H2000, 6. HBCS, 7. HRS, 8. KORA, 9. NFBC1966, 10. NTR1, 11. NTR2, 12. RS-I, 13. RS-II, 14. RS-III, 15. SardINIA, 16. SHIP, 17. THISEAS, 18. TwinsUK (pooled and female sample)/YFS (male sample), 19. YFS (pooled and female sample); A question mark indicates that the SNP was not tested in that specific study; For SNPs that were located close together in the same region, only the most significant SNP is included in the table. The last column shows the number of neighboring SNPs that exceed the threshold for suggestive SNPs.

We next attempted to replicate *in silico* the twenty suggestive SNPs in the STR (*n* = 3,271). Two of the twenty SNPs associated with self-employment were statistically significant at the 5% level in the replication study. However, the SNP effects were not in the same direction as in the majority of the discovery studies (Table S4), indicating that these SNPs were potential false positives. We then performed a combined meta-analysis of the discovery and replication studies. For all SNPs, the *p*-values were larger in the combined sample than in the discovery sample and did not reach genome-wide significance (Table S4).

The Q–Q plot for the male only meta-analysis (Figure 1B) gave a certain degree of suggestive evidence of association; however, no evidence of population stratification, cryptic relatedness, or genotyping errors was observed, as only certain SNPs–those with particularly low *p*-values–deviated from their expectation under the null hypothesis of no association. The female only meta-analysis Q–Q plot (Figure 1C) did not indicate a strong deviation for the lowest *p*-values and no evidence of population stratification, cryptic relatedness, or genotyping errors was observed. No SNPs reached genome-wide significance in the sex-stratified meta-analyses (Table 5), as can be observed in the Manhattan plots (Figures 2B and C). The male meta-analysis resulted in 22 suggestive SNPs with *p*<1×10^−5^, and the female meta-analysis resulted in sixteen suggestive SNPs (Tables 5, S5, and S6). The top SNP in males, rs6738407 (*p* = 1.52×10^−7^), was located in the *HECW2* gene, and most studies reported that carrying the minor allele decreased the probability of being self-employed. The top SNP in females, rs2331548 (*p* = 1.93×10^−6^), was located near the *CBR4* gene, and most studies estimated that carrying the minor allele decreased the probability of being self-employed.

The replication strategy for the 38 suggestive SNPs from the sex-stratified meta-analysis that were carried forward into the replication stage was similar to that used for the meta-analysis replication of the pooled data. We performed an *in silico* replication study using the data from the STR. None of the SNPs reached nominal significance (*p*<0.05) in the replication study for males only (*n* = 1,409, Table S5) and females only (*n* = 1,862, Table S6). In addition, for the majority of the suggestive SNPs, the direction of the effect was not consistently in the same direction as was reported in the majority of the discovery studies, again indicating that these SNPs were potential false positives. We meta-analyzed the results from the sex-stratified discovery meta-analysis and the replication study in a combined meta-analysis. For males, five SNPs had lower *p*-values compared to the male discovery meta-analysis, although none reached genome-wide significance (Table S5). In the combined meta-analysis for females, we observed that one SNP, rs562487, had a smaller *p*-value in this combined meta-analysis; however, this SNP did not reach genome-wide significance (*p* = 4.01×10^−6^; Table S6).

### Gene-based association analyses

The findings from the discovery meta-analyses were used to perform gene-based association tests for seventeen genes that have been previously suggested to be candidate genes for entrepreneurship [48], [79], including *ADORA2A*, *ADRA2A*, *COMT*, *DDC*, *DRD1*, *DRD2*, *DRD3*, *DRD4*, *DRD5*, *DYX1C1*, *HTR1B*, *HTR1E*, *HTR2A*, *KIAA0319* (*DYX2*), *ROBO1*, *SLC6A3* (*DAT1*), and *SNAP25*. Genes with *p*<0.003 (0.05/17 genes) were considered significant, but none of the candidate genes reached this level (Table S7).

To identify novel genes that may be associated with self-employment, we tested 17,697 genes for pooled males and females, 17,698 genes for males only, and 17,699 genes for females only, implying a significance level of *p*<2.8×10^−6^. None of the analyzed genes reached this predetermined significance level (Tables S8, S9, and S10). The gene with the lowest *p*-value was *SLC15A3* for the pooled male and female analysis (*p* = 1.63×10^−4^). For males only, the lowest *p*-value was for *TMEM156* (1.61×10^−4^), and for females only, the lowest *p*-value was for *PCP4* (*p* = 4.70×10^−5^).

We also sought to replicate the association that was reported by Nicolaou et al. [48] to exist between a common variant, rs1486011, which is located in the *DRD3* gene, and the tendency to be an entrepreneur. The SNP was nominally significant in the discovery meta-analysis (*p* = 0.011; Table S11); however, most studies reported a positive effect of the C allele–*opposite* to that reported by Nicolaou et al. [48], corroborating the results from an earlier replication study [49]. We also sought to replicate this SNP in the sex-stratified discovery meta-analyses. In this analysis, we observed a certain degree of evidence for a positive effect of the C allele in males (*p* = 0.046; Table S11) but not in females (*p* = 0.112; Table S11).

### Predicting self-employment from genotype data

We examined whether the results from the discovery meta-analyses could be used to predict self-employment in the replication study [76]. We pruned the set of autosomal SNPs to a subset of approximately 120,000 SNPs that are in approximate linkage equilibrium. In an initial prediction analysis, we included only the subset of these 120,000 SNPs that reached a 1% significance level. We calculated a predictive score for each individual in the replication study by determining, for each SNP, the product of the individual's number of effect alleles and the estimated regression coefficient from the discovery meta-analysis. This product was then summed across the included SNPs and divided by the number of included SNPs. We evaluated the predictive power of the SNPs by calculating the degree of variance in the tendency to engage in self-employment that was explained by the score and the area under the receiver operating characteristic curve (AUC). We repeated this prediction analysis eleven additional times, each time with a less stringent significance threshold required for a SNP to be included in the score. Hence, each time this analysis was performed, a larger subset of the 120,000 SNPs was analyzed.

**Figure 3:**
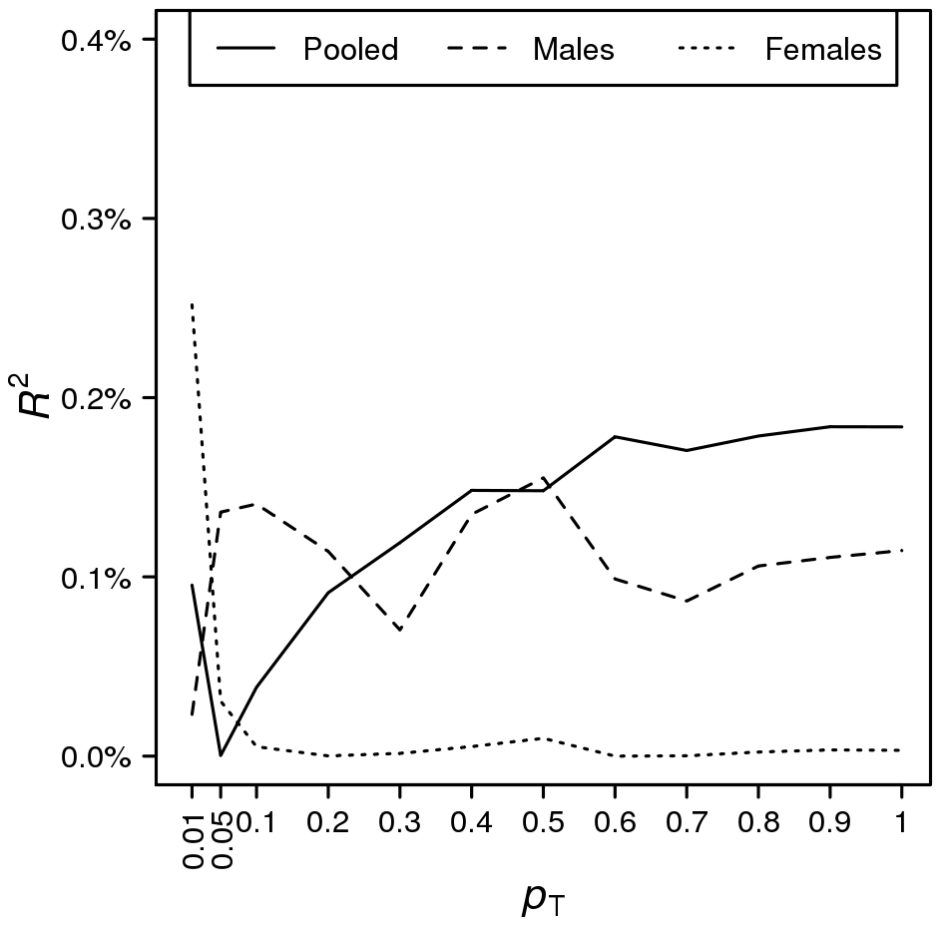
Prediction results. Variance explained (Nagelkerke pseudo-*R* ^2^ from logistic regression) vs. *p*-value threshold *p*_T_ for including SNPs in the score calculation.

For the pooled analysis of males and females (*n* = 2,589), the variance that was explained by the score reached a maximum of 0.184% when all SNPs were included (*p* = 0.039; Table S12). The scores for males only (*n* = 1,110) and for females only (*n* = 1,479) showed no evidence for association with self-employment (all *p*≥0.144, Table S12). Furthermore, we did not observe a consistent positive relationship between the variance in the tendency to engage in self-employment that was explained by the score and the significance threshold *p*_T_ (Figure 3).

## Discussion

We present results from four methods of analysis, three of which are based on genome-wide molecular genetic data, to investigate the molecular genetic architecture of self-employment.

First, using a classical twin design, we report that 55% of the variance in the tendency to engage in self-employment is due to additive genetic effects, with higher heritability for males (67%) than for females (40%). Our estimates are in agreement with those of previous twin studies. These earlier studies suggested heritabilities of 48% in a sample of primarily female British twins [26] and of 38% in a sample of US twins [28]. In addition, Zhang et al. [27] estimated the heritability of current business ownership and self-employment in a sample of Swedish twins and observed evidence of a significant additive genetic effect for females but not for males. Our results suggest significant heritability among males as well; however, the confidence intervals of the estimates are very wide for both our study and for that of Zhang et al. [27]. At least a portion of the differences between these two studies may be explained by imprecision and/or by the different samples and definitions of entrepreneurship that were used.

Second, by applying a method that was recently developed by Yang et al. [52] to entrepreneurship, we estimate that approximately 25% of the variance in the tendency to engage in self-employment (about half of the *h* ^2^ estimated in twin studies) could in principle be explained by the additive effects of common SNPs that are in linkage disequilibrium with the unknown causal variants. These results are in line with previous studies, which have estimated that common SNPs account for one-quarter to half of the narrow-sense heritability for height [52], intelligence [80], [81], personality [51], [82], several common diseases [83], schizophrenia [84], and recently for several economic and political preferences [22].

Several explanations may explain why the heritability estimate for self-employment using common SNPs is approximately half of the estimate that was obtained using the classical twin design. First, the causal variants may be in regions of the genome that are currently not covered by the available SNP arrays. Second, it is possible that the genotyped SNPs and the causal variants are not in complete linkage disequilibrium because, for example, the true causal variants have on average lower minor allele frequencies than the genotyped SNPs. Yang et al. [52] provide evidence for this in the case of human height. They estimated that 45% of the variance in height is accounted for by common SNPs, while the heritability of height is consistently estimated to be approximately 80%. The authors then developed a method that estimated the variance that was accounted for by common SNPs, assuming imperfect linkage disequilibrium between the genotyped SNPs and the unobserved causal variants. This method revealed that 84% of the variance in height, the complete heritability, could be explained by the causal variants. Twin and family studies do not suffer from this issue, as genetic relatedness is inferred from the expected relationships within the pedigree and include all of the additive genetic variation. Both of these explanations imply that the estimates that we obtained for self-employment using the more novel method are at the lower bounds of the heritability that is commonly estimated in twin and family studies. A third, alternative, explanation for the different results that were obtained using these techniques is that the twin-based heritability estimates are biased upwards because of, for example, genetic interactions [85] or a violation of the identical common environment assumption in twin studies [86].

Third, we perform the first meta-analysis of GWASs of an economic behavior (i.e., self-employment) using data from sixteen studies that together comprise approximately 50,000 participants. The discovery stage had 80% power to detect a variant at genome-wide significance with a minor allele frequency of 0.25 and odds ratios of approximately 1.11 for pooled males and females, 1.15 for males only, and 1.17 for females only [87], assuming we had a non-noisy, harmonized measure of self-employment across studies. Yet, we do not identify genome-wide significant associations. This result suggests that there are no common SNPs for self-employment with moderate to large effect sizes, thus placing an upper bound on the effect sizes of common SNPs that we can expect to exist. Gene-based tests for approximately 17,700 genes, including several candidate genes for entrepreneurship that have been previously suggested in the literature [48], [79], do not reveal significant associations. In addition, we are unable to replicate a previously reported correlation, namely, rs1486011, a SNP that is located in the *DRD3* gene. This common variant was identified by Nicolaou et al. [48], who reported its association with the tendency to be an entrepreneur. The non-replication of associations is common in candidate gene studies of human traits and behaviors. This failure to identify replicable associations is likely due to a combination of underpowered sample sizes (due to optimistic assumptions regarding plausible effect sizes) and publication bias [88]. Examples of non-replication of candidate genes studies on complex human traits include general intelligence [81], personality [89]–[94], and trust [95], [96]. We therefore stress that caution is warranted when interpreting claims from candidate gene studies of SNPs or genes with strong effects on complex behavioral traits like self-employment.

Finally, we report that a genetic score that was estimated in our meta-analysis sample has only limited predictive power in our replication study. The variance that was explained by the score was always lower than 0.26%. However, this result does not contradict our finding that approximately half of the narrow-sense heritability can be explained by common SNPs. This latter heritability analysis uses the measured SNPs to estimate realized relatedness between individuals, and given the large number of SNPs in a dense SNP array, realized relatedness can be estimated fairly accurately. In contrast, estimating a strongly predictive score from a sample requires good estimates of the effects of individual SNPs. If our discovery sample was infinitely large, it would have been possible to precisely estimate all of the SNP effects and to obtain a score with the theoretically highest possible predictive power, as estimated using the Yang et al. [52] method. The smaller the discovery sample, the noisier the estimates of the individual SNP effects; therefore, the predictive power of the score will be lower [97], [98]. Our estimates of the effects of the individual SNPs are still too imprecise to allow out-of-sample prediction with SNP data that would have practical utility.

Together, our results demonstrate that common SNPs jointly account for a substantial share of the variance in the tendency to engage in self-employment (*σ_g_* ^2^/*σ_P_* ^2^ = 25%). However, because we do not find specific SNPs in our large-scale meta-analyses of GWASs that examined self-employment, this heritability is not due to SNPs with moderate to large effects. A plausible interpretation of these results therefore appears to be that the molecular genetic architecture of self-employment is highly polygenic, implying that there are hundreds or thousands of variants that individually have a small effect and which together explain a substantial proportion of the heritability. We cannot rule out the possibility that rare genetic variants, or other, currently unmeasured, variants that are insufficiently correlated with the SNPs on the genotyping platforms, have large effects on an individual's tendency to be self-employed. However, if these genetic variants are rare, they would still not contribute a great deal to the population-based variance in self-employment, and large samples would still be required to identify these variants [51], [83], [99].

Our results are similar to those that have been reported for biologically more proximate human traits [51], [52], [80]–[82] and diseases [76], [83], [84] for which a polygenic molecular genetic architecture has also been suggested. One implication of this similarity is that, with sufficiently large sample sizes, SNPs that are associated with self-employment–and possibly also other economic variables–can in principle be discovered, as has been the case for, e.g., height [100] and BMI [101]. However, a discovery sample of approximately 50,000 individuals is apparently still too small for a meta-analysis of GWASs on a biologically distal, complex, and relatively rare human behavior such as self-employment. A potential opportunity for future research are GWASs of endophenotypes such as risk preferences, confidence, and independence. The effect sizes of individual SNPs on these endophenotypes may be larger because of their greater biological proximity. However, these variables are difficult to measure reliably and not (yet) available in many genotyped samples.

Given the need for very large samples in meta-analyses of GWASs on complex traits, an important challenge of the present study was to identify a measure of entrepreneurship that is available in a sufficiently large sample. We opted to maximize the available sample size in this study and operationalized entrepreneurship as self-employment, which is also the most frequently used measure of entrepreneurship in the economics literature [102].

We included every study we were aware of in the analysis that included a measure of self-employment and which was willing to contribute data, although this approach necessitated that data from diverse populations (e.g., Eastern German self-employed individuals and US business owners) were pooled. The available measures of self-employment varied across studies, including different single- and multiple-item measures, data from stand-alone surveys, and data from repeated measures or retrospective employment histories of the participants. For a number of studies, this approach resulted in a lack of detailed and reliable data regarding work-life history. Substantial measurement error, especially with respect to the definition of the control group, was therefore unavoidable. Ideally, the control group would encompass only participants who had never been self-employed and who will never be self-employed. Such an analysis would have required data regarding the complete work-life history of participants and participants who had reached an appropriate age. However, only data regarding current employment status were available in the majority of the contributing studies. It is therefore possible that there was a certain degree of misclassification in the studies that included only single-item, single-response measures of self-employment, thereby adding noise to the phenotype definition and potentially reducing the statistical power with respect to association detection.

Statistical power may have also been reduced by heterogeneity within the case group, as this group comprised individuals who became self-employed for very different reasons. For example, certain individuals may have chosen self-employment because they had no viable alternatives in paid employment, whereas others may have done so because of their desire to pursue a business opportunity. The motivations, goals, and resources of these two groups of individuals are obviously very different, and the genetics underlying these various characteristics may likewise differ greatly. Unfortunately, more detailed information regarding the motivations, activities, and success of entrepreneurs was unavailable for most of the genotyped samples.

In general, GWASs face a practical trade-off between phenotype quality and sample size. Surprisingly, statistical power calculations suggest that studying a more noisy phenotype in a larger sample is often more likely to be successful than studying a perfect phenotype in a small sample. For example, assume that a common SNP exists with a minor allele frequency of 0.5 that increases the odds for all types of entrepreneurship by a factor of 1.13 on average (assuming 15% of the population are entrepreneurs and the data are population samples). The required sample size to detect this SNP with 80% power for a perfectly-measured outcome is approximately 30,000. Measuring entrepreneurship perfectly would require a lengthier survey that is administered more than once. Such a large genotyped sample with perfect measures of entrepreneurship does not currently exist. Smaller samples with perfect measures would be underpowered to detect the SNP. In contrast, if the available measures for entrepreneurship are noisy and have a test-retest reliability of only 0.6-which is typical for behavioral traits measured by brief surveys [103]–[105]−80% power to detect this SNP requires a discovery sample of approximately 50,000 individuals. Thus, our study was well-powered to detect effects of this magnitude even if there was substantial measurement error and noise in the data.

The results of our study have three implications for this future research agenda. First, the high share of variance in self-employment that can be attributed towards interpersonal differences in common SNPs suggests that this research agenda is in principle feasible. Second, to investigate if and how genes that are related to economic variables influence medical outcomes, it will be necessary in the future to identify either the specific genetic variants that are underlying the heritability of economic variables (i.e., to investigate causal pathways from genes to medical outcomes), or to calculate genetic scores that have at least moderate out-of-sample predictive power (i.e., to investigate the medical consequences of a mismatch between genetic predisposition and economic outcomes). Even larger samples than what we had available in our present study will be needed to identify genome-wide significant SNPs and to estimate more accurate genetic scores for economic variables. Third, our results suggest that the effects of single SNPs on self-employment are likely to be very small. Given these effect sizes, statistical power calculations suggests that a research strategy that aims to maximize sample size by pooling data with slightly inaccurate measures of self-employment is more likely to be successful than a research strategy that aims to collect perfect phenotype measures in a much smaller sample. If successful, this research could shed new light on the complex interaction of genes, environment, and personal choices on health and longevity.

## Supporting Information

Table S1 **Study design, sample size, sample quality control, and self-employment measure within each study.**
(DOC)

Table S2 **Genotyping, imputation, SNP quality control, and statistical analysis within each study.**
(DOC)

Table S3 **Genomic inflation factors.**
(DOC)

Table S4 **Replication results of the twenty suggestive SNPs (p<1×10−5) from the self-employment discovery meta-analyses for pooled males and females.**
(DOC)

Table S5 **Replication results of the 22 suggestive SNPs (p<1×10−5) from the self-employment discovery meta-analyses for males only.**
(DOC)

Table S6 **Replication results of the sixteen suggestive SNPs (p<1×10−5) from the self-employment discovery meta-analyses for females only.**
(DOC)

Table S7 **Gene-based p-values for the candidate entrepreneurship genes for pooled males and females, males only, and females only.**
(DOC)

Table S8 **Gene-based p-values for the top 25 genes associated with self-employment in the discovery meta-analysis for pooled males and females.**
(DOC)

Table S9 **Gene-based p-values for the top 25 genes associated with self-employment in the discovery meta-analysis for males only.**
(DOC)

Table S10 **Gene-based p-values for the top 25 genes associated with self-employment in the discovery meta-analysis for females only.**
(DOC)

Table S11 **Meta-analysis association results for SNP rs1486011 for pooled males and females, males only, and females only.**
(DOC)

Table S12 **Results of the prediction analyses in STR for pooled males and females, males only, and females only.**
(DOC)
