# Supplementary material for: The Molecular Genetic Architecture of Self-Employment
Source: PLoS One. 2013 Apr 4;8(4):e60542. doi: 10.1371/journal.pone.0060542 (PMC3617140; doi:10.1371/journal.pone.0060542)
Supplement: Table S1 — Study design, sample size, sample quality control, and self-employment measure within each study. (DOC) [file pone.0060542.s001.doc]

**Table S1. Study design, sample size, sample quality control, and self-employment measure within each study.**

| **Study** | |  |  | **Sample QC** | |  |  |  |
| --- | --- | --- | --- | --- | --- | --- | --- | --- |
| **Abbreviation** | **Full name** | **Study design** | **Total sample size (*n*)** | **Call rate** | **Other exclusions** | **Sample size after QC (*n*)** | **Self-employment measure** | **References** |
| AGES | Age, Gene/Environment Susceptibility–Reykjavik Study | Population-based | 3,219 | ≥ 98% | 1. Mismatch previous genotypes; 2. gender discrepancy; 3. missing self-employment status. | 3,219 | Questionnaire: Are you a employer or manager? | 1. Harris TB, Launer LJ, Eiriksdottir G, Kjartansson O, Jonsson PV, et al. (2007) Age, Gene/Environment Susceptibility–Reykjavik Study: Multidisciplinary applied phenomics. Am J Epidemiol 165: 1076–1087. |
| ASPS | Austrian Stroke Prevention Study | Population-based | 2,008 | ≥ 98% | 1. Excess autosomal heterozygosity; 2. mismatch between called and phenotypic gender; 3. outliers in the IBD analysis; 4. missing self-employment status. | 834 | Participants were systematically asked about their life-long professional activities and this information is recorded in the subjects’ charts. In addition information was gathered as to whether the subjects conducted their professional activities as employees or self-employed. | 1. Schmidt R, Lechner H, Fazekas F, Niederkorn K, Reinhart B, et al. (1994) Assessment of cerebrovascular risk proles in healthy persons: denition of research goals and the Austrian Stroke Prevention Study (ASPS). Neuroepidemiology 13: 308–313. |
| ERF | Erasmus Rucphen Family study | Family- based | 3,500 | ≥ 98% | 1. Heterozygosity: FDR<1%; 2. ethnic outliers; 3. duplicates; 4. gender mismatch; 5. excess IBS incompatible with pedigree; 6. missing self-employment status. | 1,071 | Individuals were asked if they were ever self-employed (no; yes—once; yes—more than once; always; not applicable/no response) and coded as self-employed if they were self-employed at at least than once. The control group consists of people who were never self-employed. | 1. Pardo LM, MacKay I, Oostra B, van Duijn CM, Aulchenko YS (2005) The effect of genetic drift in a young genetically isolated population. Ann Hum Genet 69: 288–295. |
| GHS | Gutenberg Health Study | Population-based | 3,500 | ≥ 97% | 1. Deviations in expected heterozygosity; 2. doubtful IBS patterns; 3. missing self-employment status. | 3,130 | Categorization of the actual job or last job (for participants who retired); questions on each job phase of the professional career were collected in a computer-assisted personal interview (CAPI). | 1. Rotival M, Zeller T, Wild PS, Maouche S, Szymczak S, et al. (2011) Integrating genome-wide genetic variations and monocyte expression data reveals trans-regulated gene modules in humans. PLoS Genet 7: e1002367. |
| H2000 | Health 2000 | Population-based | 8,028 | ≥ 95% | 1. Excess heterozygosity; 2. relatedness and/or failed gender check; 3. missing self-employment status. | 2,123 | Participants were asked “Are you/were you 1) Salary earner, 2) Agricultural entrepreneur, 3) Other entrepreneur, 4) Self-employed person or freelancer, 5) Working in a family member's farm without a salary, 6) Working in a family member's business without a salary, 7) Other, 8) Never been working in a full time job, 9) don't know” Salary earners were coded as controls and particpants that answered 2,3 or 4 were coded as cases. Other participants were excluded. | 1. Aromaa A, Koskinen,S. (Eds) (2004). Health and functional capacity in Finland. Publications of the National Public Health Institute, KTL B12: Helsinki: Finland. |
| HBCS | Helsinki Birth Cohort Study | Birth cohort study | 8,760 | ≥ 95% | 1. Excess heterozygosity; 2. relatedness and/or failed gender check; 3. missing self-employment status. | 1,724 | In the HBCS, data on classification of the socio-economic status and self-employment were derived from the Statistics Finland. These data were available with five-year-interval from 1970 to 2000. Participant were classified as self-employed if at any of the time points they indicated self-employment. Participants were excluded if data in more than two (out of seven) time points were missing (n = 4). In addition, those controls with indication of self-employment were excluded. | 1. Eriksson JG (2005) Early growth and adult health outcomes–lessons learned from the Helsinki Birth Cohort Study. Matern Child Nutr 1: 149–154. |
| HRS | Health and Retirement Study | Population-based | 12,507 | ≥ 98% | 1. Relatedness check; 2. ethnic outliers; 3. missing self-employment status. | 6,220 | From the HRS RAND v.L dataset the binary variables r*slfemp were used that indicate if an individual was self-employed or working for someone else in wave *. Individuals are coded as self-employed if they responded in at least one data wave to be self-employed. The control group consists of people who were never self-employed but indicated at least once to work for someone else. | 1. Weir D (2007) Elastic Powers: The Integration of Biomarkers into the Health and Retirement Study, Washington, D.C, USA: The National Academies Press, chapter Biosocial Surveys. pp. 78–95. |
| KORA S4 | Cooperative Health Research in the Region of Augsburg | Population-based | 4,261 | > 93% | 1. Gender mismatch; 2. missing self-employment status. | 1,724 | Questionnaire based. “Which position do you/did you have in your job?” | 1. Wichmann HE, Gieger C, Illig T, MONICA/KORA Study Group (2005) Kora-gen–resource for population genetics, controls and a broad spectrum of disease phenotypes. Gesundheitswesen 67 Suppl 1: S26–S30. |
| NFBC1966 | Northern Finland Birth Cohort 1966 | Population-based | 12,231 | ≥ 95% | 1. Gender discrepancy with genetic data from X-linked markers; 2. withdrawn consent; 3. duplicates and first and second degree relatives; 4. contaminated samples. 5. missing self-employment status. | 4,234 | Based on questionnaire questions Q1-Q3, the subjects were classified into 9 groups according to the instructions from Statistics Finland (Reference: Tilastokeskus Käsikirjoja 17: Sosioekonomisen aseman luokitus 1989): 1) Farm businessmen 2) Other enterpreneuers 3) Upper white-collar workers 4) Lower white-collar workers 5) Blue-collar workers 6) Students 7) Pensioners 8) Unemployed (incl. long-term unemployed and unclassified) 9) Socio-economic status unknown. For the present analysis groups 1) and 2) are considered as cases and others as controls, excluding group 7) pensioners and from group 8) long-term unemployed and unclassified. | 1. Rantakallio P (1969) Groups at risk in low birth weight infants and perinatal mortality. Acta Paediatr Scand 193: Suppl 193:1–71 2. Sabatti C, Service SK, Hartikainen AL, Pouta A, Ripatti S, et al. (2009) Genome-wide association analysis of metabolic traits in a birth cohort from a founder population. Nat Genet 41: 35–46. |
| NTR1 | Netherlands Twin Register Cohort 1 | Twin study | 29,852 | > 90% | 1. Presence of genetic data; 2. gender discrepancy with genetic data; 3. unexpected IBS sharing; 4. contaminated samples; 5. duplicates and first and second degree relatives; 6. missing self-employment status. | 1,555 | Data came from eight surveys. Participants were asked to indicate whether they were self-employed (1991, 1993, 1995, 2004, 2009) or to indicate the type of organisation that they worked in, with being self-employed as one of the answer categories (1997, 2000, 2002). If they indicated to be self-employed in any one of the surveys, they were classified as self-employed. | 1. Boomsma DI, de Geus EJC, Vink JM, Stubbe JH, Distel MA, et al. (2006) Netherlands Twin Register: From twins to twin families. Twin Res Hum Genet 9: 849–857. 2. Boomsma DI, Willemsen G, Sullivan PF, Heutink P, Meijer P, et al. (2008) Genome-wide association of major depression: description of samples for the GAIN Major Depressive Disorder Study: NTR and NESDA biobank projects. Eur J Hum Genet 16: 335–342. |
| NTR2 | Netherlands Twin Register Cohort 2 | Twin study | 29,852 | > 90% | 1. Presence of genetic data; 2. gender discrepancy with genetic data; 3. unexpected IBS sharing; 4. contaminated samples; 5. duplicates and first and second degree relatives; 6. missing self-employment status. | 984 | Data came from eight surveys. Participants were asked to indicate whether they were self-employed (1991, 1993, 1995, 2004, 2009) or to indicate the type of organisation that they worked in, with being self-employed as one of the answer categories (1997, 2000, 2002). If they indicated to be self-employed in any one of the surveys, they were classified as self-employed. | 1. Willemsen G, de Geus EJC, Bartels M, van Beijsterveldt CEMT, Brooks AI, et al. (2010) The Netherlands Twin Register biobank: A resource for genetic epidemiological studies. Twin Res Hum Genet 13: 231–245. |
| RS-I | Rotterdam Study Baseline | Population-based | 7,983 | ≥ 97.5% | 1. Gender mismatch with typed Xlinked markers; 2. excess autosomal heterozygosity > 0.336~FDR>0.1%; 3. duplicates and/or 1st or 2nd degree relatives using IBS probabilities >97% from PLINK; 4. ethnic outliers using IBS distances > 3SD from PLINK; 5. missing self-employment status. | 5,374 | Detailed background information on the entire work-life history of all participants was available, including the number and duration of self-employment spells. Participants who had at least one spell in self-employment were coded as cases, those with zero spells as controls. Participants with incomplete work-life histories were excluded. | 1. Hofman A, van Duijn CM, Franco OH, Ikram MA, Janssen HLA, et al. (2011) The rotterdam study: 2012 objectives and design update. Eur J Epidemiol 26: 657–686. 2. Estrada K, Abuseiris A, Grosveld FG, Uitterlinden AG, Knoch TA, et al. (2009) Grimp: a web- and grid-based tool for high-speed analysis of large-scale genome-wide association using imputed data. Bioinformatics 25: 2750–2752. |
| RS-II | Rotterdam Study Extension of Baseline | Population-based | 3,011 | ≥ 97.5% | 1. Gender mismatch with typed Xlinkedmarkers; 2. excess autosomal heterozygosity (F<-0.055); 3. duplicates and/or 1st degree relatives using IBD PiHAT >40% from PLINK; 4. ethnic outliers IBS distances > 4SD mean HaMAP CEU cluster from PLINK; 5. missing self-employment status. | 2,066 | Information on current employment status was available and self-employed participants were coded as cases. Participants that indicated employment or that were collaborating family members were coded as controls. Other participants were excluded. | 1. Hofman A, van Duijn CM, Franco OH, Ikram MA, Janssen HLA, et al. (2011) The rotterdam study: 2012 objectives and design update. Eur J Epidemiol 26: 657–686. 2. Estrada K, Abuseiris A, Grosveld FG, Uitterlinden AG, Knoch TA, et al. (2009) Grimp: a web- and grid-based tool for high-speed analysis of large-scale genome-wide association using imputed data. Bioinformatics 25: 2750–2752. |
| RS-III | Rotterdam Study Young | Population-based | 3,932 | ≥ 97.5% | 1. Gender mismatch with typed Xlinked markers; 2. excess autosomal heterozygosity (F<-0.055); 3. duplicates and/or 1st degree relatives using IBD PiHAT >40% from PLINK; 4. ethnic outliers IBS distances > 4SD mean HaMAP CEU cluster from PLINK; 5. missing self-employment status. | 1,925 | Information on current or last employment status (if retired) was available and self-employed participants were coded as cases. Participants that indicated employment or that were collaborating family members were coded as controls. Other participants were excluded. | 1. Hofman A, van Duijn CM, Franco OH, Ikram MA, Janssen HLA, et al. (2011) The rotterdam study: 2012 objectives and design update. Eur J Epidemiol 26: 657–686. 2. Estrada K, Abuseiris A, Grosveld FG, Uitterlinden AG, Knoch TA, et al. (2009) Grimp: a web- and grid-based tool for high-speed analysis of large-scale genome-wide association using imputed data. Bioinformatics 25: 2750–2752. |
| SardINIA | SardiNIA Study of Aging | Population-based | 6,148 | ≥ 95% | 1. missing genotype; 2. missing self-employment status. | 4,142 | Information on current employment status was available and self-employed participants were coded as cases. | 1. Pilia G, Chen WM, Scuteri A, Orr M, Albai G, et al. (2006) Heritability of cardiovascular and personality traits in 6,148 Sardinians. PLoS Genet 2: e132. |
| SHIP | Study of Health in Pomerania | Population-based | 4,308 | ≥ 92% | 1. Duplicate samples (by IBS); 2. reported/genotyped gender mismatch; 3. missing self-employment status. | 4,063 | “Which occupational position do you currently have?” Participants were coded as self-employed if they answered that they were farmers with more than 10 hectare property, university graduates with a liberal profession (physician, lawyer, tax accountant, etc.) or self-employed (in business, craft, or the tertiary sector). | 1. Völzke H, Alte D, Schmidt CO, Radke D, Lorbeer R, et al. (2011) Cohort profile: The Study of Health in Pomerania. Int J Epidemiol 40: 294–307. |
| STR | Swedish Twin Registry | Twin study | 10,946 | ≥ 97% | 1. Sex-check (heterozygosity of X-chomosomes); 2. deviations in heterozygosity of more then 5 SD from the population mean; 3. cryptically relatedness check; 4. missing self-employment status. | 3,271 | Participants were asked “(1) have you ever run a business?” and “(2) how many businesses have you in total been part of starting?” Participants were coded as cases if they answered “Yes” to the first and “≥1” to the second question. Controls were those that answered “No” to the first and “0” to the second question. Other participants were excluded. | 1. Lichtenstein P, Sullivan PF, Cnattingius S, Gatz M, Johansson S, et al. (2006) The Swedish Twin Registry in the third millennium: An update. Twin Res Hum Genet 9: 875–882. 2. Cesarini D, Johannesson M, Magnusson PKE, Wallace B (2011) The behavioral genetics of behavioral anomalies. Manage Sci: In press. |
| THISEAS | The Hellenic study of Interactions between SNPs & Eating in Atherosclerosis Susceptibility | CAD case-control | 1,877 | > 95% | 1. Missing self-employment status; 2. heterozygosity; 3. gender mismatch; 4. ethnic outliers. | 685 | Information on current employment status was available. Participants were asked whether they were: 1) civil servant, 2) private employee, 3) self-employed, 4)part-time employee, 5) retired, or 6) house holding. Self-employed participants were coded as cases. Participants who were retired were excluded. | 1. Theodoraki EV, Nikopensius T, Suhorutsenko J, Peppes V, Fili P, et al. (2010) Fibrinogen beta variants confer protection against coronary artery disease in a Greek case-control study. BMC Med Genet 11: 28. |
| TwinsUK | The UK Adult Twin Registry | Twin study | 4,427 | ≥ 95% | 1. Heterozygosity across all SNPs ≥2 s.d. from the sample mean; 2. evidence of non‐European ancestry as assessed by PCA comparison with HapMap3 populations; 3. observed pairwise IBD probabilities suggestive of sample identity errors; 4. missing self-employment status. | 3,155 | Information on the duration of self-employment was available. Particpants that indicated to have spent some time in self-employment were coded as cases, those that indicated to have never been self-employed were coded as controls. Other participants were excluded. | 1. Moayyeri A, Hammond CJ, Valdes AM, Spector TD (2012) Cohort profile: TwinsUK and healthy ageing twin study. Int J Epidemiol: In press. 2. Aulchenko YS, Ripke S, Isaacs A, van Duijn CM (2007) GenABEL: An R library for genome-wide association analysis. Bioinformatics 23: 1294–1296. |
| YFS | The Cardiovascular Risk in Young Finns Study | Population-based cohort | 3,596 | ≥ 95% | 1. Excess heterozygosity; 2. relatedness and/or failed gender check. | 2,358 | Study subjects were asked in years 1986, 1989, 1992 and 2001 if they were 1) Salary earners 2) Farm businessmen 3) Unpaid workers at their family farm 4) Entrepreneurs 5) Unpaid workers at their family business or 6) Others. Persons who indicated to be farm businessmen or entrepreneurs in any of the surveys were classified as cases. Those who hadn’t in any time point reported to be an entrepreneur or farm businessman were selected as controls. | 1. Raitakari OT, Juonala M, Rönnemaa T, Keltikangas-Järvinen L, Räsänen L, et al. (2008). Cohort profile: The cardiovascular risk in Young Finns Study. Int J Epidemiol 37: 1220–1226. |
