# Supplementary material for: The Molecular Genetic Architecture of Self-Employment
Source: PLoS One. 2013 Apr 4;8(4):e60542. doi: 10.1371/journal.pone.0060542 (PMC3617140; doi:10.1371/journal.pone.0060542)
Supplement: Table S2 — Genotyping, imputation, SNP quality control, and statistical analysis within each study. (DOC) [file pone.0060542.s002.doc]

Table S2. Genotyping, imputation, SNP quality control, and statistical analysis within each study.

|  | **Genotyping** | | | | | | **Imputation and quality control before meta-analsis** | | | | **Association analysis** | |
| --- | --- | --- | --- | --- | --- | --- | --- | --- | --- | --- | --- | --- |
|  |  |  | **SNP inclusion criteria** | | |  |  | **SNP inclusion criteria** | |  |  |  |
| **Study** | **Platform(s), SNP panel(s)** | **Genotyping calling algorithm** | **MAF** | **Call rate** | **HWE** | **Genotyped SNPs after QC** | **Imputation software** | **MAF** | **Imputation quality** | **Imputed SNPs after QC** | **Software** | **Covariates** |
| AGES | Illumina Human370CNV | BeadStudio | ≥ 1% | ≥ 98% | ≥ 10^-6^ | 317,344 | MACH | ≥ 1% | Rsq ≥ 0.4 | 2,176,303 | ProbABEL | 1. Sex in pooled sample; 2. first four PCs in all samples; 3. age dummies for the categories ≤ 29 (reference), 30–39, 40–49, ≥ 50. |
| ASPS | Illumina Human610-Quad BeadChip | BeadStudio | ≥ 1% | ≥ 98% | ≥ 10^-6^ | 550,635 | MACH | ≥ 5% | Rsq ≥ 0.4 | 2,164,654 | GenABEL | 1. Sex in pooled sample; 2. first four PCs. |
| ERF | Illumina 6K, 318K, 370K, Affymetrix 250K, Illumina 610K | Beadstudio using Hap300v2 cluster file | ≥ 1% | ≥ 98% | ≥ 10^-6^ | 650,197 | MACH | ≥ 1% | Rsq ≥ 0.4 | 2,353,164 | ProbABEL | 1. Sex in pooled sample; 2. age dummies for the categories ≤ 29 (reference), 30–39, 40–49, ≥ 50. |
| GHS | Affymetrix 6.0 | Birdseed | ≥ 1% | ≥ 98% | ≥ 10^-4^ | 649,182 | IMPUTE | ≥ 5% | info ≥ 0.4 | 2,220,912 | SNPTEST | 1. Sex in pooled sample; 2. age dummies for the categories ≤ 29 (reference), 30–39, 40–49, ≥ 50 |
| Health 2000 | Illumina Human610-Quad BeadChip | Illuminus | ≥ 1% | ≥ 95% | ≥ 10^-6^ | 555,418 | MACH | ≥ 1% | Rsq ≥ 0.4 | 2,463,699 | ProbABEL | 1. Sex in pooled sample; 2. first four PCs; 3. age dummies for the categories ≤ 29 (reference), 30–39, 40–49, ≥ 50. |
| HBCS | Illumina Human660-Quad BeadChip | Illuminus | ≥ 1% | ≥ 95% | ≥ 10^-6^ | 533,491 | MACH | ≥ 1% | Rsq ≥ 0.4 | 2,416,556 | ProbABEL | 1. Sex in pooled sample; 2. first four PCs in all samples. |
| HRS | Illumina Omni2.5 Beadchip | GenomeStudio | ≥ 1% | ≥ 98% | ≥ 10^-4^ | 2,195,306 | MACH | ≥ 1% | Rsq ≥ 0.4 | 2,227,690 | PLINK | 1. Sex in pooled sample; 2. first four PCs in all samples; 3. age dummies for the categories 30–39 (reference), 40–49, ≥ 50. |
| KORA S4 | Affymetrix 6.0 | Birdseed2 | — | — | — | 909,622 | IMPUTE | ≥ 1% | info ≥ 0.4 | 2,521,850 | SNPTEST | 1. Sex in pooled sample; 2. age categories (≤ 39; 40–49; ≥ 50). |
| NFBC1966 | Illumina HumanCNV-370DUO Analysis BeadChip | Beadstudio | ≥ 5% | ≥ 95% | ≥ 10^-4^ | 328,007 | IMPUTE | ≥ 1% | info ≥ 0.4 | 2,405,775 | SNPTEST | 1. Sex in pooled sample; 2. first four PCs. |
| NTR1 | Perlegen–Affymetrix 600K | Affymetrix Proprietary | ≥ 1% | ≥ 95% | > 10^-5^ | 427,049 | IMPUTE | ≥ 1% | info ≥ 0.4 | 2,420,149 | SNPTEST | 1. Sex in pooled sample; 2. age dummies for the categories ≤ 29 (reference), 30–39, 40–49, ≥ 50. |
| NTR2 | IlluminaTM Human660W-QuadXX | Beadstudio | > 1% | > 95% | > 10^-5^ | 528,072 | IMPUTE | ≥ 1% | info ≥ 0.4 | 2,532,400 | SNPTEST | 1. Sex in pooled sample; 2. age dummies for the categories ≤ 29 (reference), 30–39, 40–49, ≥ 50. |
| RS-I | Illumina HumanHap 550 V.3 | BeadStudio Genecall | ≥ 1% | ≥ 98% | ≥ 10^-6^ | 512,349 | MACH | ≥ 1% | Rsq ≥ 0.4 | 2,433,150 | MACH2DAT | 1. Sex in pooled sample; 2. first four PCs. |
| RS-II | Illumina HumanHap 550 V.3 DUO; Illumina HumanHap 610 QUAD | Genomestudio Genecall | ≥ 1% | ≥ 97.5% | ≥ 10^-6^ | 466,389 | MACH | ≥ 1% | Rsq ≥ 0.4 | 2,432,613 | MACH2DAT | 1. Sex in pooled sample; 2. first four PCs. |
| RS-III | Illumina HumanHap 610 QUAD | Genomestudio Genecall | ≥ 1% | ≥ 97.5% | ≥ 10^-6^ | 514,073 | MACH | ≥ 1% | Rsq ≥ 0.4 | 2,436,797 | MACH2DAT | 1. Sex in pooled sample; 2. first four PCs; 3. dummy for age ≥ 50. |
| SardINIA | Affymetrix 10k, 500k, 1M | BRLMM | ≥ 5% | ≥ 95% | ≥ 10^-6^ | 356,359 | MACH | ≥ 5% | Rsq ≥ 0.4 | 1,972,533 | Merlin | 1. Sex in pooled sample. |
| SHIP | Affymetrix Human SNP Array 6.0 | Birdseed V2 | — | — | — | 869,224 | IMPUTE | ≥ 1% | info ≥ 0.4 | 2,514,047 | QUICKTEST | 1) Sex in pooled sample;  2) age dummies for the categories ≤ 29 (reference), 30–39, 40–49, ≥ 50. |
| STR | Illumina HumanOmniExpress BeadChip | GenomeStudio Genecall | ≥ 1% | ≥ 97% | ≥ 10^-7^ | 644,556 | IMPUTE | ≥ 1% | info ≥ 0.4 | 2,481,423 | Merlin | 1. Sex in pooled sample; 2. first four PCs. |
| THISEAS | CardioMetaboChip Array | GenoSNP | ≥ 5% | ≥ 95% | ≥ 10^-6^ | 96,015 | — | ≥ 1% | — | 95,510^a^ | PLINK | 1. age dummies for the categories ≤ 29 (reference), 30–39, 40–49, ≥ 50. |
| TwinsUK | Illumina Human610-Quad BeadChip | Illuminus | ≥ 5% | ≥ 95% | ≥ 10^-5^ | 536,559 | IMPUTE | ≥ 1% | info ≥ 0.4 | 2,262,054 | GenABEL | 1. Sex in pooled sample; 2. first four PCs; 3. age dummies for the categories ≤ 29 (reference), 30–39, 40–49, ≥ 50. |
| YFS | Illumina Human670-Quad Custom BeadChip | Illuminus | ≥ 1% | ≥ 95% | ≥ 10^-6^ | 546,677 | MACH | ≥ 1% | Rsq ≥ 0.4 | 2,409,746 | ProbABEL | 1. Sex in pooled sample; 2. first four PCs in all samples; 3. age dummies for the categories ≤ 29 (reference), 30-39, 40-49, ≥ 50. |

^a^ Number of genotyped SNPs after filtering on minor allele frequency ≥ 1%.
