# Supplementary material for: The Molecular Genetic Architecture of Self-Employment
Source: PLoS One. 2013 Apr 4;8(4):e60542. doi: 10.1371/journal.pone.0060542 (PMC3617140; doi:10.1371/journal.pone.0060542)
Supplement: Table S3 — Genomic inflation factors. (DOC) [file pone.0060542.s003.doc]

**Table S3. Genomic inflation factors*.***

| **Study** | **Pooled** | **Males** | **Females** |
| --- | --- | --- | --- |
| AGES | 1.000 | 1.019 | 0.992 |
| ASPS | 1.117 | 0.998 | 0.998 |
| ERF | 1.105 | 1.099 | 1.042 |
| GHS | 0.996 | 1.006 | 0.999 |
| H2000 | 0.990 | 0.994 | 0.995 |
| HBCS | 1.005 | 0.995 | 1.007 |
| HRS | 1.008 | 1.006 | 0.998 |
| KORA S4 | 1.000 | 1.008 | 1.001 |
| NFBC1966 | 1.003 | 1.007 | 1.004 |
| NTR1 | 1.006 | 1.015 | 1.002 |
| NTR2 | 1.004 | 1.021 | 1.022 |
| RS-I | 1.022 | 1.008 | 1.003 |
| RS-II | 1.008 | 1.015 | 0.998 |
| RS-III | 1.000 | 1.004 | 1.013 |
| SardINIA | 1.074 | 1.038 | 1.156 |
| SHIP | 1.010 | 1.021 | 0.989 |
| STR | 0.996 | 0.997 | 0.990 |
| THISEAS^a^ | — | — | — |
| TwinsUK^b^ | 1.015 | — | 1.011 |
| YFS | 1.010 | 1.015 | 1.007 |

^a^ THISEAS did not provide results using genome-wide SNP data and genomic control lambdas were therefore not estimated.

^b^ The number of male subjects was insufficient for a male stratified analysis.
