# Supplementary material for: The Molecular Genetic Architecture of Self-Employment
Source: PLoS One. 2013 Apr 4;8(4):e60542. doi: 10.1371/journal.pone.0060542 (PMC3617140; doi:10.1371/journal.pone.0060542)
Supplement: Table S4 — Replication results of the twenty suggestive SNPs (p<1×10^−5^) from the self-employment discovery meta-analyses for pooled males and females. (DOC) [file pone.0060542.s004.doc]

**Table S4. Replication results of the twenty suggestive SNPs (*p* < 1 × 10^-5^) from the self-employment discovery meta-analyses for pooled males and females.**

| **Discovery meta-analysis** | | | | | | | | | | | **Swedish Twin Registry** | | **Combined meta-analysis** | | |
| --- | --- | --- | --- | --- | --- | --- | --- | --- | --- | --- | --- | --- | --- | --- | --- |
| **SNP** | **Chr.** | **Pos.** | **Effect / non-effect allele** | **Nearest gene** | **Distance to gene (bp)** | ***I*^2^** | **Cochran's *Q* test *p*-value** | ***n*** | ***p*-value** | **Overall freq.** | ***p*-value** | **Freq.** | ***p*-value** | **Direction** | **Improvement?** |
| rs6906622 | 6 | 18,596,287 | T/C | RNF144B | 19,460 | 0.0 | 0.644 | 49,942 | 4.10 × 10^-6^ | 0.21 | 0.30 | 0.19 | 2.54 × 10^-5^ | ++-++++++++-++++?++- | no |
| rs477588 | 6 | 18,586,095 | T/C | RNF144B | 9,268 | 0.0 | 0.646 | 49,942 | 4.15 × 10^-6^ | 0.21 | 0.32 | 0.19 | 2.46 × 10^-5^ | ++-++++++++-++++?++- | no |
| rs9350075 | 6 | 18,595,091 | A/C | RNF144B | 18,264 | 0.0 | 0.640 | 49,942 | 4.26 × 10^-6^ | 0.79 | 0.31 | 0.81 | 2.63 × 10^-5^ | --+--------+----?--+ | no |
| rs3920593 | 6 | 18,595,112 | T/C | RNF144B | 18,285 | 0.0 | 0.641 | 49,942 | 4.35 × 10^-6^ | 0.79 | 0.30 | 0.81 | 2.68 × 10^-5^ | --+--------+----?--+ | no |
| rs6928004 | 6 | 18,597,621 | A/G | RNF144B | 20,794 | 0.0 | 0.640 | 49,942 | 4.47 × 10^-6^ | 0.79 | 0.31 | 0.81 | 2.71 × 10^-5^ | --+--------+----?--+ | no |
| rs4716268 | 6 | 18,598,474 | A/C | RNF144B | 21,647 | 0.0 | 0.639 | 49,942 | 4.60 × 10^-6^ | 0.21 | 0.31 | 0.19 | 2.76 × 10^-5^ | ++-++++++++-++++?++- | no |
| rs10776614 | 10 | 49,433,172 | T/C | ARHGAP22 | 49,972 | 0.0 | 0.980 | 49,942 | 4.79 × 10^-6^ | 0.16 | 0.78 | 0.17 | 6.78 × 10^-6^ | -+-----+--------?--- | no |
| rs2358531 | 5 | 75,515,542 | A/G | SV2C | 100,482 | 0.0 | 0.660 | 46,812 | 4.79 × 10^-6^ | 0.71 | 0.01 | 0.72 | 1.42 × 10^-4^ | ---?------+--+--?--+ | no |
| rs747533 | 10 | 49,426,264 | A/G | ARHGAP22 | 56,880 | 0.0 | 0.973 | 49,942 | 5.26 × 10^-6^ | 0.26 | 0.92 | 0.26 | 1.14 × 10^-5^ | -+-----+--------?--+ | no |
| rs510367 | 6 | 18,591,491 | A/G | RNF144B | 14,664 | 0.0 | 0.677 | 49,942 | 5.33 × 10^-6^ | 0.21 | 0.31 | 0.19 | 3.20 × 10^-5^ | ++-++++++++-++++?++- | no |
| rs6899634 | 6 | 18,600,965 | A/C | RNF144B | 24,138 | 0.0 | 0.619 | 49,942 | 5.54 × 10^-6^ | 0.21 | 0.32 | 0.19 | 3.19 × 10^-5^ | ++-++++++++-++++?++- | no |
| rs17166082 | 7 | 131,363,900 | A/G | PLXNA4 | 94,730 | 0.0 | 0.821 | 39,758 | 5.82 × 10^-6^ | 0.06 | 0.88 | 0.05 | 9.37 × 10^-6^ | -?-?--?--+------?--- | no |
| rs994208 | 14 | 33,531,622 | C/G | EGLN3 | 41,587 | 0.0 | 0.741 | 49,942 | 6.11 × 10^-6^ | 0.66 | 0.62 | 0.68 | 2.05 × 10^-5^ | -+--------------?--+ | no |
| rs3847697 | 12 | 57,282,257 | T/C | LRIG3 | 269,947 | 0.0 | 0.762 | 41,566 | 6.79 × 10^-6^ | 0.44 | 0.64 | 0.43 | 2.19 × 10^-5^ | --+-----?-+-+-?-?--+ | no |
| rs9371065 | 6 | 18,601,109 | A/T | RNF144B | 24,282 | 0.0 | 0.682 | 49,942 | 8.84 × 10^-6^ | 0.22 | 0.32 | 0.19 | 4.84 × 10^-5^ | ++-++++++++-++++?++- | no |
| rs2057556 | 6 | 18,602,356 | T/C | RNF144B | 25,529 | 0.0 | 0.693 | 49,942 | 8.98 × 10^-6^ | 0.78 | 0.33 | 0.81 | 4.88 × 10^-5^ | --+--------+----?--+ | no |
| rs10514062 | 5 | 75,513,972 | A/T | SV2C | 98,912 | 0.0 | 0.636 | 49,942 | 8.99 × 10^-6^ | 0.72 | 0.01 | 0.72 | 2.44 × 10^-4^ | ----------+--+--?--+ | no |
| rs3742467 | 14 | 49,709,284 | T/C | SOS2 | 55,689 | 3.5 | 0.413 | 43,722 | 9.11 × 10^-6^ | 0.88 | 0.44 | 0.89 | 3.97 × 10^-5^ | +++++-?+-+++-+++?++- | no |
| rs1324564 | 6 | 18,603,428 | T/C | RNF144B | 26,601 | 0.0 | 0.698 | 49,942 | 9.21 × 10^-6^ | 0.78 | 0.33 | 0.81 | 4.97 × 10^-5^ | --+--------+----?--+ | no |
| rs1359339 | 6 | 18,606,507 | T/G | RNF144B | 29,680 | 0.0 | 0.775 | 49,942 | 9.77 × 10^-6^ | 0.22 | 0.33 | 0.19 | 5.24 × 10^-5^ | ++-++++++++-++++?++- | no |

Chr.: chromosome; Pos.: position; Overall freq.: average effect allele frequency; In the column “direction”, the studies are in the following order: 1. AGES, 2. ASPS, 3. ERF, 4. GHS, 5. H2000, 6. HBCS, 7. HRS, 8. KORA S4, 9. NFBC1966, 10. NTR1, 11. NTR2, 12. RS-I, 13. RS-II, 14. RS-III, 15. SardINIA, 16. SHIP, 17. THISEAS, 18. TwinsUK, 19. YFS, 20. STR; A question mark indicates that the SNP was not tested in that specific study.
