# Supplementary material for: The Molecular Genetic Architecture of Self-Employment
Source: PLoS One. 2013 Apr 4;8(4):e60542. doi: 10.1371/journal.pone.0060542 (PMC3617140; doi:10.1371/journal.pone.0060542)
Supplement: Table S5 — Replication results of the 22 suggestive SNPs (p<1×10^−5^) from the self-employment discovery meta-analyses for males only. (DOC) [file pone.0060542.s005.doc]

**Table S5. Replication results of the 22 suggestive SNPs (*p* < 1 × 10^-5^) from the self-employment discovery meta-analyses for males only.**

| **Discovery meta-analysis** | | | | | | | | | | | **Swedish Twin Registry** | | **Combined meta-analysis** | | |
| --- | --- | --- | --- | --- | --- | --- | --- | --- | --- | --- | --- | --- | --- | --- | --- |
| **SNP** | **Chr.** | **Pos.** | **Effect / non-effect allele** | **Nearest gene** | **Distance to gene (bp)** | ***I*^2^** | **Cochran's *Q* test *p*-value** | ***n*** | ***p*-value** | **Overall freq.** | ***p*-value** | **Freq.** | ***p*-value** | **Direction** | **Improvement?** |
| rs6738407 | 2 | 196,851,876 | A/G | HECW2 | 79,655 | 0.0 | 0.819 | 21,508 | 1.52 × 10^-7^ | 0.20 | 0.26 | 0.20 | 7.93 × 10^-8^ | ---------+------?-- | yes |
| rs7558876 | 2 | 196,884,193 | T/C | HECW2 | 111,972 | 0.0 | 0.676 | 21,508 | 2.02 × 10^-7^ | 0.25 | 0.98 | 0.26 | 4.85 × 10^-7^ | ---------++-----?-+ | no |
| rs4850390 | 2 | 196,851,234 | A/T | HECW2 | 79,013 | 0.0 | 0.807 | 21,508 | 2.18 × 10^-7^ | 0.20 | 0.26 | 0.20 | 1.14 × 10^-7^ | ---------+------?-- | yes |
| rs12619045 | 2 | 196,883,649 | A/G | HECW2 | 111,428 | 0.0 | 0.707 | 21,508 | 2.41 × 10^-7^ | 0.75 | 0.97 | 0.74 | 5.81 × 10^-7^ | +++++++++--+++++?+- | no |
| rs17829296 | 2 | 196,871,827 | A/G | HECW2 | 99,606 | 0.0 | 0.643 | 21,508 | 2.75 × 10^-7^ | 0.80 | 0.77 | 0.81 | 9.04 × 10^-7^ | +++++++-++++++++?+- | no |
| rs1869794 | 2 | 196,880,651 | T/G | HECW2 | 108,430 | 0.0 | 0.650 | 21,508 | 2.82 × 10^-7^ | 0.20 | 0.81 | 0.19 | 8.72 × 10^-7^ | -------+--------?-+ | no |
| rs4145361 | 2 | 196,849,516 | T/C | HECW2 | 77,295 | 0.0 | 0.767 | 21,508 | 3.94 × 10^-7^ | 0.80 | 0.27 | 0.80 | 2.12 × 10^-7^ | +++++++++-++++++?++ | yes |
| rs11889112 | 2 | 196,873,346 | A/G | HECW2 | 101,125 | 0.0 | 0.624 | 21,508 | 4.23 × 10^-7^ | 0.20 | 0.81 | 0.19 | 1.27 × 10^-6^ | -------+--------?-+ | no |
| rs11901261 | 2 | 196,874,778 | A/C | HECW2 | 102,557 | 0.0 | 0.623 | 21,508 | 4.28 × 10^-7^ | 0.80 | 0.81 | 0.81 | 1.28 × 10^-6^ | +++++++-++++++++?+- | no |
| rs11902488 | 2 | 196,875,256 | A/G | HECW2 | 103,035 | 0.0 | 0.623 | 21,508 | 4.31 × 10^-7^ | 0.80 | 0.81 | 0.81 | 1.29 × 10^-6^ | +++++++-++++++++?+- | no |
| rs10190368 | 2 | 196,870,691 | A/G | HECW2 | 98,470 | 0.0 | 0.618 | 21,508 | 4.50 × 10^-7^ | 0.80 | 0.87 | 0.81 | 1.22 × 10^-6^ | +++++++-++++++++?+- | no |
| rs6757097 | 2 | 196,882,699 | T/C | HECW2 | 110,478 | 0.0 | 0.705 | 21,508 | 4.87 × 10^-7^ | 0.25 | 0.95 | 0.26 | 1.16 × 10^-6^ | ---------++-----?-+ | no |
| rs2889155 | 2 | 196,852,550 | T/G | HECW2 | 80,329 | 0.0 | 0.704 | 18,680 | 5.27 × 10^-7^ | 0.82 | 0.61 | 0.82 | 6.10 × 10^-7^ | ++++++?-++++++++?++ | no |
| rs2124384 | 2 | 196,877,435 | T/C | HECW2 | 105,214 | 0.0 | 0.646 | 19,786 | 5.49 × 10^-7^ | 0.80 | 0.83 | 0.81 | 1.58 × 10^-6^ | +++++++-++++++?+?+- | no |
| rs12473229 | 2 | 196,878,519 | T/G | HECW2 | 106,298 | 0.0 | 0.681 | 18,680 | 9.25 × 10^-7^ | 0.75 | 0.94 | 0.74 | 2.16 × 10^-6^ | ++++++?++--+++++?+- | no |
| rs2305567 | 2 | 196,881,061 | A/G | HECW2 | 108,840 | 0.0 | 0.690 | 21,508 | 9.84 × 10^-7^ | 0.75 | 0.94 | 0.74 | 2.30 × 10^-6^ | +++++++++--+++++?+- | no |
| rs6730618 | 2 | 196,865,163 | T/C | HECW2 | 92,942 | 0.0 | 0.720 | 21,508 | 3.34 × 10^-6^ | 0.78 | 0.92 | 0.79 | 5.89 × 10^-6^ | +-+++++++++-++++?++ | no |
| rs6825440 | 4 | 183,636,063 | A/T | ODZ3 | 153,933 | 24.1 | 0.175 | 21,508 | 4.25 × 10^-6^ | 0.24 | 0.07 | 0.21 | 9.68 × 10^-7^ | -+--------+---+-?-- | yes |
| rs7904494 | 10 | 72,056,694 | A/T | PRF1 | 24,157 | 15.2 | 0.280 | 18,680 | 6.74 × 10^-6^ | 0.78 | 0.32 | 0.80 | 3.79 × 10^-5^ | +-+---?--++-----?-+ | no |
| rs1378626 | 2 | 196,864,466 | T/C | HECW2 | 92,245 | 0.0 | 0.667 | 21,508 | 7.62 × 10^-6^ | 0.21 | 0.54 | 0.20 | 7.10 × 10^-6^ | -+---------+----?-- | yes |
| rs4867424 | 5 | 32,331,331 | T/C | MTMR12 | 17,540 | 0.0 | 0.919 | 21,508 | 8.39 × 10^-6^ | 0.49 | 0.83 | 0.50 | 2.00 × 10^-5^ | --+-------------?-+ | no |
| rs2712008 | 4 | 38,752,396 | T/G | KLHL5 | 11,942 | 0.0 | 0.815 | 18,680 | 9.94 × 10^-6^ | 0.14 | 0.44 | 0.11 | 4.26 × 10^-5^ | +-++++?+++++-+++?+- | no |

Chr.: chromosome; Pos.: position; Overall freq.: average effect allele frequency; In the column “direction”, the studies are in the following order: 1. AGES, 2. ASPS, 3. ERF, 4. GHS, 5. H2000, 6. HBCS, 7. HRS, 8. KORA S4, 9. NFBC1966, 10. NTR1, 11. NTR2, 12. RS-I, 13. RS-II, 14. RS-III, 15. SardINIA, 16. SHIP, 17. THISEAS, 18. YFS, 19. STR; A question mark indicates that the SNP was not tested in that specific study.
