# Supplementary material for: The Molecular Genetic Architecture of Self-Employment
Source: PLoS One. 2013 Apr 4;8(4):e60542. doi: 10.1371/journal.pone.0060542 (PMC3617140; doi:10.1371/journal.pone.0060542)
Supplement: Table S6 — Replication results of the sixteen suggestive SNPs (p<1×10^−5^) from the self-employment discovery meta-analyses for females only. (DOC) [file pone.0060542.s006.doc]

**Table S6. Replication results of the sixteen suggestive SNPs (*p* < 1 × 10^-5^) from the self-employment discovery meta-analyses for females only.**

| **Discovery meta-analysis** | | | | | | | | | | | **Swedish Twin Registry** | | **Combined meta-analysis** | | |
| --- | --- | --- | --- | --- | --- | --- | --- | --- | --- | --- | --- | --- | --- | --- | --- |
| **SNP** | **Chr.** | **Pos.** | **Effect / non-effect allele** | **Nearest gene** | **Distance to gene (bp)** | ***I*^2^** | **Cochran's *Q* test *p*-value** | ***n*** | ***p*-value** | **Overall freq.** | ***p*-value** | **Freq.** | ***p*-value** | **Direction** | **Improvement?** |
| rs2331548 | 4 | 170,199,179 | A/G | CBR4 | 31,182 | 0.0 | 0.913 | 24,319 | 1.93 × 10^-6^ | 0.96 | 0.95 | 0.96 | 4.30 × 10^-6^ | ??+?++++++++++++?++- | no |
| rs521326 | 6 | 52,927,336 | A/G | GSTA4 | 23,373 | 0.0 | 0.558 | 28,174 | 2.92 × 10^-6^ | 0.61 | 0.48 | 0.63 | 1.33 × 10^-5^ | ------------+---?--+ | no |
| rs1022335 | 2 | 145,813,253 | A/T | ZEB2 | 818,867 | 0.0 | 0.762 | 24,782 | 3.02 × 10^-6^ | 0.37 | 0.48 | 0.36 | 1.37 × 10^-5^ | ------?-----+---?--+ | no |
| rs487642 | 6 | 52,926,012 | A/G | GSTA4 | 24,697 | 0.0 | 0.557 | 28,174 | 3.05 × 10^-6^ | 0.39 | 0.47 | 0.38 | 1.42 × 10^-5^ | ++++++++++++-+++?++- | no |
| rs575861 | 6 | 52,931,798 | A/C | GSTA4 | 18,911 | 0.0 | 0.599 | 28,174 | 3.83 × 10^-6^ | 0.61 | 0.50 | 0.63 | 1.63 × 10^-5^ | ------------+---?--+ | no |
| rs10753804 | 1 | 168,583,032 | T/C | SCYL1BP1 | 184,861 | 0.0 | 0.848 | 24,782 | 3.92 × 10^-6^ | 0.49 | 0.98 | 0.42 | 7.97 × 10^-6^ | ------?----+----?--+ | no |
| rs521664 | 6 | 52,930,496 | T/C | GSTA4 | 20,213 | 0.0 | 0.593 | 28,174 | 3.95 × 10^-6^ | 0.61 | 0.53 | 0.63 | 1.60 × 10^-5^ | ------------+---?--+ | no |
| rs594614 | 6 | 52,927,410 | A/C | GSTA4 | 23,299 | 0.0 | 0.528 | 28,440 | 4.15 × 10^-6^ | 0.39 | 0.48 | 0.38 | 1.83 × 10^-5^ | ++++++++++++-+++-++- | no |
| rs562487 | 5 | 78,442,190 | A/G | BHMT | 1,169 | 0.0 | 0.630 | 28,174 | 4.49 × 10^-6^ | 0.48 | 0.50 | 0.50 | 4.01 × 10^-6^ | +++++-++-+-++-++?+++ | yes |
| rs9557259 | 13 | 99,031,403 | T/C | TM9SF2 | 18,125 | 0.0 | 0.592 | 12,506 | 5.16 × 10^-6^ | 0.06 | 0.34 | 0.03 | 2.92 × 10^-5^ | ??-?++?++++++?????+- | no |
| rs1383043 | 4 | 123,562,066 | A/G | ADAD1 | 8,323 | 24.9 | 0.167 | 25,279 | 6.05 × 10^-6^ | 0.38 | 0.71 | 0.35 | 1.78 × 10^-5^ | --+----+--------??++ | no |
| rs9578700 | 13 | 23,775,308 | A/G | SPATA13 | 740 | 38.9 | 0.047 | 28,174 | 6.53 × 10^-6^ | 0.67 | 0.34 | 0.70 | 3.65 × 10^-5^ | -+++------------?-++ | no |
| rs4481907 | 1 | 168,599,170 | T/C | SCYL1BP1 | 168,723 | 0.0 | 0.835 | 28,174 | 7.88 × 10^-6^ | 0.51 | 0.99 | 0.58 | 1.48 × 10^-5^ | +++++++++++-++++?+++ | no |
| rs947230 | 13 | 23,772,926 | A/C | SPATA13 | 3,122 | 40.1 | 0.041 | 28,174 | 8.02 × 10^-6^ | 0.67 | 0.33 | 0.70 | 4.39 × 10^-5^ | -+++------------?-++ | no |
| rs7684834 | 4 | 123,260,318 | A/G | KIAA1109 | 50,889 | 31.7 | 0.097 | 28,174 | 8.69 × 10^-6^ | 0.62 | 0.82 | 0.65 | 1.27 × 10^-5^ | ++-++++-++++++++?--+ | no |
| rs557302 | 5 | 78,413,090 | A/G | BHMT2 | 7,939 | 0.0 | 0.716 | 28,174 | 8.77 × 10^-6^ | 0.49 | 0.69 | 0.49 | 1.06 × 10^-5^ | -----+--+-+-----?--- | no |

Chr.: chromosome; Pos.: position; Overall freq.: average effect allele frequency; In the column “direction”, the studies are in the following order: 1. AGES, 2. ASPS, 3. ERF, 4. GHS, 5. H2000, 6. HBCS, 7. HRS. 8. KORA S4, 9. NFBC1966, 10. NTR1, 11. NTR2, 12. RS-I, 13. RS-II, 14. RS-III, 15. SardINIA, 16. SHIP, 17. THISEAS, 18. TwinsUK, 19. YFS, 20. STR; A question mark indicates that the SNP was not tested in that specific study
