# Supplementary material for: The Molecular Genetic Architecture of Self-Employment
Source: PLoS One. 2013 Apr 4;8(4):e60542. doi: 10.1371/journal.pone.0060542 (PMC3617140; doi:10.1371/journal.pone.0060542)
Supplement: Table S7 — Gene-based p-values for the candidate entrepreneurship genes for pooled males and females, males only, and females only. (DOC) [file pone.0060542.s007.doc]

**Table S7. Gene-based *p*-values for the candidate entrepreneurship genes for pooled males and females, males only, and females only.**

| **Gene** | **Pooled** | **Males** | **Females** |
| --- | --- | --- | --- |
| ADORA2A | 0.228 | 0.464 | 0.293 |
| ADRA2A | 0.007 | 0.011 | 0.183 |
| COMT | 0.528 | 0.999 | 0.073 |
| DDC | 0.334 | 0.758 | 0.604 |
| DRD1 | 0.666 | 0.331 | 0.366 |
| DRD2 | 0.749 | 0.843 | 0.786 |
| DRD3 | 0.012 | 0.010 | 0.603 |
| DRD4 | 0.483 | 0.366 | 0.221 |
| DRD5 | 0.689 | 0.803 | 0.417 |
| DYX1C1 | 0.384 | 0.164 | 0.347 |
| HTR1B | 0.892 | 0.975 | 0.511 |
| HTR1E | 0.953 | 0.518 | 0.597 |
| HTR2A | 0.079 | 0.030 | 0.685 |
| KIAA0319 (DYX2) | 0.324 | 0.477 | 0.419 |
| ROBO1 | 0.554 | 0.692 | 0.435 |
| SLC6A3 (DAT1) | 0.679 | 0.645 | 0.627 |
| SNAP25 | 0.118 | 0.209 | 0.888 |
