# Supplementary material for: The Molecular Genetic Architecture of Self-Employment
Source: PLoS One. 2013 Apr 4;8(4):e60542. doi: 10.1371/journal.pone.0060542 (PMC3617140; doi:10.1371/journal.pone.0060542)
Supplement: Table S8 — Gene-based p-values for the top 25 genes associated with self-employment in the discovery meta-analysis for pooled males and females. (DOC) [file pone.0060542.s008.doc]

**Table S8. Gene-based *p*-values for the top 25 genes associated with self-employment in the discovery meta-analysis for pooled males and females.**

| **Chr.** | **Gene** | **Number of SNPs** | **Start position** | **Stop position** | ***p*-value** |
| --- | --- | --- | --- | --- | --- |
| 11 | SLC15A3 | 78 | 60,461,135 | 60,475,833 | 1.63 × 10^-4^ |
| 11 | TMEM132A | 67 | 60,448,488 | 60,461,207 | 2.46 × 10^-4^ |
| 11 | PRPF19 | 78 | 60,414,777 | 60,430,632 | 2.61 × 10^-4^ |
| 11 | TMEM109 | 68 | 60,438,252 | 60,447,491 | 2.73 × 10^-4^ |
| 11 | CD6 | 109 | 60,495,690 | 60,544,424 | 3.05 × 10^-4^ |
| 9 | FANCG | 65 | 35,063,834 | 35,070,013 | 3.15 × 10^-4^ |
| 6 | FBXL4 | 132 | 99,428,321 | 99,502,570 | 3.31 × 10^-4^ |
| 9 | PIGO | 68 | 35,078,687 | 35,086,579 | 3.51 × 10^-4^ |
| 7 | SLC26A5 | 112 | 102,780,412 | 102,873,834 | 3.53 × 10^-4^ |
| 11 | ZP1 | 73 | 60,391,590 | 60,399,740 | 3.84 × 10^-4^ |
| 9 | DNAJB5 | 62 | 34,979,784 | 34,988,428 | 4.02 × 10^-4^ |
| 9 | VCP | 68 | 35,046,064 | 35,062,739 | 4.03 × 10^-4^ |
| 9 | C9orf131 | 59 | 35,031,101 | 35,035,988 | 5.02 × 10^-4^ |
| 9 | KIAA1539 | 68 | 35,094,117 | 35,105,893 | 5.05 × 10^-4^ |
| 9 | STOML2 | 67 | 35,089,888 | 35,093,154 | 5.55 × 10^-4^ |
| 14 | C14orf138 | 90 | 49,645,099 | 49,653,047 | 5.63 × 10^-4^ |
| 9 | KIAA1045 | 76 | 34,948,191 | 34,972,541 | 6.03 × 10^-4^ |
| 10 | SHOC2 | 95 | 112,713,902 | 112,763,413 | 6.81 × 10^-4^ |
| 5 | PRLR | 293 | 35,099,984 | 35,266,334 | 7.03 × 10^-4^ |
| 6 | IHPK3 | 205 | 33,797,420 | 33,822,660 | 7.24 × 10^-4^ |
| 14 | LOC196913 | 102 | 49,620,118 | 49,629,111 | 1.00 × 10^-3^ |
| 14 | SOS2 | 159 | 49,653,595 | 49,767,849 | 1.02 × 10^-3^ |
| 6 | C6orf125 | 181 | 33,773,323 | 33,787,482 | 1.05 × 10^-3^ |
| 19 | TMEM190 | 41 | 60,580,015 | 60,581,424 | 1.22 × 10^-3^ |
| 15 | TMOD2 | 138 | 49,831,101 | 49,889,635 | 1.26 × 10^-3^ |
