# Supplementary material for: The Molecular Genetic Architecture of Self-Employment
Source: PLoS One. 2013 Apr 4;8(4):e60542. doi: 10.1371/journal.pone.0060542 (PMC3617140; doi:10.1371/journal.pone.0060542)
Supplement: Table S9 — Gene-based p-values for the top 25 genes associated with self-employment in the discovery meta-analysis for males only. (DOC) [file pone.0060542.s009.doc]

**Table S9. Gene-based *p*-values for the top 25 genes associated with self-employment in the discovery meta-analysis for males only.**

| **Chr.** | **Gene** | **Number of SNPs** | **Start position** | **Stop position** | ***p*-value** |
| --- | --- | --- | --- | --- | --- |
| 4 | TMEM156 | 171 | 38,644,835 | 38,710,436 | 1.61 × 10^-4^ |
| 4 | KLHL5 | 146 | 38,723,053 | 38,800,224 | 3.21 × 10^-4^ |
| 11 | SLCO2B1 | 139 | 74,539,810 | 74,594,947 | 3.57 × 10^-4^ |
| 5 | STARD4 | 96 | 110,861,920 | 110,876,056 | 4.19 × 10^-4^ |
| 8 | TMEM67 | 50 | 94,836,268 | 94,899,523 | 4.48 × 10^-4^ |
| 17 | TNFSF12-TNFSF13 | 63 | 7,393,139 | 7,405,649 | 4.67 × 10^-4^ |
| 1 | OR2M2 | 89 | 246,409,910 | 246,410,954 | 4.71 × 10^-4^ |
| 4 | ING2 | 74 | 184,663,213 | 184,669,243 | 4.73 × 10^-4^ |
| 17 | SAT2 | 47 | 7,470,280 | 7,471,889 | 4.98 × 10^-4^ |
| 17 | TNFSF12 | 61 | 7,393,098 | 7,401,931 | 5.16 × 10^-4^ |
| 17 | TNFSF13 | 49 | 7,402,339 | 7,405,642 | 5.16 × 10^-4^ |
| 17 | EIF4A1 | 44 | 7,416,780 | 7,423,048 | 5.18 × 10^-4^ |
| 17 | SENP3 | 46 | 7,406,042 | 7,416,011 | 5.29 × 10^-4^ |
| 1 | OR2M5 | 75 | 246,375,072 | 246,376,011 | 6.39 × 10^-4^ |
| 2 | HECW2 | 496 | 196,772,221 | 197,165,580 | 7.41 × 10^-4^ |
| 17 | CD68 | 44 | 7,423,528 | 7,426,153 | 7.41 × 10^-4^ |
| 17 | SHBG | 48 | 7,474,215 | 7,477,395 | 8.32 × 10^-4^ |
| 1 | OR2M3 | 98 | 246,432,992 | 246,433,931 | 8.35 × 10^-4^ |
| 17 | SOX15 | 46 | 7,432,221 | 7,434,212 | 9.16 × 10^-4^ |
| 17 | MPDU1 | 48 | 7,427,853 | 7,432,247 | 1.00 × 10^-3^ |
| 2 | PAX3 | 205 | 222,772,850 | 222,871,944 | 1.02 × 10^-3^ |
| 8 | RBM12B | 35 | 94,812,903 | 94,822,400 | 1.07 × 10^-3^ |
| 15 | TMOD2 | 139 | 49,831,101 | 49,889,635 | 1.10 × 10^-3^ |
| 17 | FXR2 | 58 | 7,435,271 | 7,458,796 | 1.22 × 10^-3^ |
| 19 | TMEM190 | 42 | 60,580,015 | 60,581,424 | 1.28 × 10^-3^ |
