# Supplementary material for: The Molecular Genetic Architecture of Self-Employment
Source: PLoS One. 2013 Apr 4;8(4):e60542. doi: 10.1371/journal.pone.0060542 (PMC3617140; doi:10.1371/journal.pone.0060542)
Supplement: Table S10 — Gene-based p-values for the top 25 genes associated with self-employment in the discovery meta-analysis for females only. (DOC) [file pone.0060542.s010.doc]

**Table S10. Gene-based *p*-values for the top 25 genes associated with self-employment in the discovery meta-analysis for females only.**

| **Chr.** | **Gene** | **Number of SNPs** | **Start position** | **Stop position** | ***p*-value** |
| --- | --- | --- | --- | --- | --- |
| 21 | PCP4 | 227 | 40,161,216 | 40,223,192 | 4.70 × 10^-5^ |
| 9 | MELK | 94 | 36,562,904 | 36,667,679 | 2.02 × 10^-4^ |
| 2 | FLJ20160 | 155 | 190,981,325 | 191,075,286 | 2.48 × 10^-4^ |
| 5 | BHMT2 | 107 | 78,401,338 | 78,421,031 | 2.66 × 10^-4^ |
| 4 | ADAD1 | 62 | 123,519,617 | 123,570,389 | 2.98 × 10^-4^ |
| 4 | KIAA1109 | 93 | 123,311,207 | 123,503,357 | 3.76 × 10^-4^ |
| 4 | IL2 | 47 | 123,592,075 | 123,597,100 | 3.81 × 10^-4^ |
| 5 | BHMT | 74 | 78,443,359 | 78,463,869 | 4.26 × 10^-4^ |
| 15 | CSPG4 | 54 | 73,753,717 | 73,792,244 | 5.15 × 10^-4^ |
| 4 | IL21 | 106 | 123,753,232 | 123,761,661 | 5.46 × 10^-4^ |
| 5 | ACTBL2 | 128 | 56,811,599 | 56,814,393 | 6.98 × 10^-4^ |
| 5 | ACTBL2 | 128 | 56,811,599 | 56,814,393 | 7.33 × 10^-4^ |
| 8 | PCMTD1 | 194 | 52,892,692 | 52,974,288 | 7.53 × 10^-4^ |
| 15 | SNX33 | 36 | 73,728,402 | 73,738,023 | 8.43 × 10^-4^ |
| 15 | ODF3L1 | 37 | 73,803,373 | 73,807,082 | 9.80 × 10^-4^ |
| 15 | ODF3L1 | 37 | 73,803,373 | 73,807,082 | 9.90 × 10^-4^ |
| 2 | HIBCH | 203 | 190,777,604 | 190,892,804 | 1.09 × 10^-3^ |
| 2 | GKN2 | 121 | 69,025,867 | 69,033,606 | 1.15 × 10^-3^ |
| 22 | C22orf30 | 74 | 30,402,241 | 30,438,731 | 1.16 × 10^-3^ |
| 5 | C5orf35 | 92 | 56,240,859 | 56,248,770 | 1.17 × 10^-3^ |
| 6 | STX11 | 120 | 144,513,346 | 144,554,769 | 1.17 × 10^-3^ |
| 6 | GPX6 | 59 | 28,579,051 | 28,591,549 | 1.32 × 10^-3^ |
| 2 | INPP1 | 110 | 190,916,440 | 190,944,636 | 1.34 × 10^-3^ |
| 7 | SKAP2 | 333 | 26,673,212 | 26,870,866 | 1.44 × 10^-3^ |
| 2 | MGC13057 | 140 | 190,710,730 | 190,776,455 | 1.58 × 10^-3^ |
