# Supplementary material for: The Molecular Genetic Architecture of Self-Employment
Source: PLoS One. 2013 Apr 4;8(4):e60542. doi: 10.1371/journal.pone.0060542 (PMC3617140; doi:10.1371/journal.pone.0060542)
Supplement: Table S11 — Meta-analysis association results for SNP rs1486011 for pooled males and females, males only, and females only. (DOC) [file pone.0060542.s011.doc]

**Table S11. Meta-analysis association results for SNP rs1486011 for pooled males and females, males only, and females only.**

| **Sample** | **Effect / non-effect allele** | **EAF** | ***n*** | ***p*-value** | **Direction** |
| --- | --- | --- | --- | --- | --- |
| Pooled | C/G | 0.074 | 45,800 | 0.011 | +++++++-+--++-?+?-+ |
| Males | C/G | 0.077 | 19,786 | 0.046 | +-++++--+--+-+?+?+ |
| Females | C/G | 0.072 | 25,754 | 0.112 | +--++++--++++-?+?-+ |

EAF: average allele frequency; In the column “direction”, the studies are in the following order: 1. AGES, 2. ASPS, 3. ERF, 4. GHS, 5. H2000, 6. HBCS, 7. HRS, 8. KORA S4, 9. NFBC1966, 10. NTR1, 11. NTR2, 12. RS-I, 13. RS-II, 14. RS-III, 15. SardINIA, 16. SHIP, 17. THISEAS, 18. TwinsUK (pooled and female sample) / YFS (male sample), 19. YFS (pooled and female sample); A question mark indicates that the SNP was not tested in that specific study.
