# Supplementary material for: The Molecular Genetic Architecture of Self-Employment
Source: PLoS One. 2013 Apr 4;8(4):e60542. doi: 10.1371/journal.pone.0060542 (PMC3617140; doi:10.1371/journal.pone.0060542)
Supplement: Table S12 — Results of the prediction analyses in STR for pooled males and females, males only, and females only. (DOC) [file pone.0060542.s012.doc]

**Table S12. Results of the prediction analyses in STR for pooled males and females, males only, and females only.**

|  | **Pooled** | | | | **Males** | | | | **Females** | | | |
| --- | --- | --- | --- | --- | --- | --- | --- | --- | --- | --- | --- | --- |
| **Threshold** | **Coeff.** | ***p*-value** | ***R*^2^ (%)** | **AUC** | **Coeff.** | ***p*-value** | ***R*^2^ (%)** | **AUC** | **Coeff.** | ***p*-value** | ***R*^2^ (%)** | **AUC** |
| p_T_ < 0.01 | -0.293 | 0.898 | 0.095% | 0.518 | -0.088 | 0.667 | 0.023% | 0.515 | -0.424 | 0.924 | 0.252% | 0.530 |
| p_T_ < 0.05 | -0.005 | 0.531 | 0.000% | 0.490 | 0.055 | 0.148 | 0.136% | 0.507 | -0.037 | 0.692 | 0.031% | 0.508 |
| p_T_ < 0.1 | 0.076 | 0.210 | 0.038% | 0.517 | 0.086 | 0.144 | 0.141% | 0.511 | 0.023 | 0.418 | 0.005% | 0.498 |
| p_T_ < 0.2 | 0.183 | 0.107 | 0.091% | 0.519 | 0.123 | 0.170 | 0.114% | 0.513 | -0.006 | 0.513 | 0.000% | 0.500 |
| p_T_ < 0.3 | 0.277 | 0.078 | 0.119% | 0.521 | 0.127 | 0.226 | 0.070% | 0.512 | -0.027 | 0.545 | 0.002% | 0.508 |
| p_T_ < 0.4 | 0.382 | 0.057 | 0.148% | 0.524 | 0.221 | 0.149 | 0.135% | 0.519 | 0.062 | 0.417 | 0.005% | 0.506 |
| p_T_ < 0.5 | 0.460 | 0.057 | 0.148% | 0.524 | 0.285 | 0.132 | 0.155% | 0.520 | 0.100 | 0.388 | 0.010% | 0.519 |
| p_T_ < 0.6 | 0.591 | 0.041 | 0.178% | 0.526 | 0.266 | 0.187 | 0.099% | 0.517 | 0.000 | 0.500 | 0.000% | 0.500 |
| p_T_ < 0.7 | 0.067 | 0.045 | 0.170% | 0.523 | 0.286 | 0.203 | 0.087% | 0.516 | 0.018 | 0.485 | 0.000% | 0.497 |
| p_T_ < 0.8 | 0.077 | 0.041 | 0.179% | 0.525 | 0.360 | 0.178 | 0.106% | 0.518 | 0.007 | 0.446 | 0.002% | 0.513 |
| p_T_ < 0.9 | 0.088 | 0.039 | 0.184% | 0.525 | 0.041 | 0.173 | 0.111% | 0.520 | 0.010 | 0.433 | 0.003% | 0.512 |
| p_T_ ≤ 1.0 | 0.098 | 0.039 | 0.184% | 0.525 | 0.047 | 0.169 | 0.115% | 0.520 | 0.011 | 0.435 | 0.003% | 0.516 |

Prediction results are based on a logistic regression of self-employment on the score controlling for the number of non-missing genotypes. Approximately 120,000 directly genotyped SNPs were used to calculate the score in STR for 12 overlapping significance thresholds. The R^2^ is the Nagelkerke pseudo-R^2^ from the logistic regression. The p-value indicates the significance of the score coefficient (one-sided Wald test, since the alternative hypothesis is that the score is positively correlated with self-employment). The AUC is the area under the receiver operating characteristic curve.
